# Early-Onset and Robust Amyloid Pathology in a New Homozygous Mouse Model of Alzheimer's Disease

**DOI:** 10.1371/journal.pone.0007931

**Published:** 2009-11-20

**Authors:** Antje Willuweit, Joachim Velden, Robert Godemann, Andre Manook, Fritz Jetzek, Hartmut Tintrup, Gunther Kauselmann, Branko Zevnik, Gjermund Henriksen, Alexander Drzezga, Johannes Pohlner, Michael Schoor, John A. Kemp, Heinz von der Kammer

**Affiliations:** 1 Evotec Neurosciences GmbH, Hamburg, Germany; 2 Nuklearmedizinische Klinik und Poliklinik, Klinikum rechts der Isar, Technische Universität München, Munich, Germany; 3 Evotec Technologies GmbH, Hamburg, Germany; 4 TaconicArtemis GmbH, Cologne, Germany; National Institutes of Health, United States of America

## Abstract

**Background:**

Transgenic mice expressing mutated amyloid precursor protein (APP) and presenilin (PS)-1 or -2 have been successfully used to model cerebral β-amyloidosis, one of the characteristic hallmarks of Alzheimer's disease (AD) pathology. However, the use of many transgenic lines is limited by premature death, low breeding efficiencies and late onset and high inter-animal variability of the pathology, creating a need for improved animal models. Here we describe the detailed characterization of a new homozygous double-transgenic mouse line that addresses most of these issues.

**Methodology/Principal Findings:**

The transgenic mouse line (ARTE10) was generated by co-integration of two transgenes carrying the K670N/M671L mutated amyloid precursor protein (APP_swe_) and the M146V mutated presenilin 1 (PS1) both under control of a neuron-specific promoter. Mice, hemi- as well as homozygous for both transgenes, are viable and fertile with good breeding capabilities and a low rate of premature death. They develop robust AD-like cerebral β-amyloid plaque pathology with glial inflammation, signs of neuritic dystrophy and cerebral amyloid angiopathy. Using our novel image analysis algorithm for semi-automatic quantification of plaque burden, we demonstrate an early onset and progressive plaque deposition starting at 3 months of age in homozygous mice with low inter-animal variability and 100%-penetrance of the phenotype. The plaques are readily detected in vivo by PiB, the standard human PET tracer for AD. In addition, ARTE10 mice display early loss of synaptic markers and age-related cognitive deficits. By applying a γ-secretase inhibitor we show a dose dependent reduction of soluble amyloid β levels in the brain.

**Conclusions:**

ARTE10 mice develop a cerebral β-amyloidosis closely resembling the β-amyloid-related aspects of human AD neuropathology. Unifying several advantages of previous transgenic models, this line particularly qualifies for the use in target validation and for evaluating potential diagnostic or therapeutic agents targeting the amyloid pathology of AD.

## Introduction

Alzheimer's disease (AD), the most common form of dementia in the elderly, is characterized by extracellular deposition of amyloid plaques, the intracellular aggregation of neurofibrillary tangles (NFTs) and loss of synaptic connections in the brain. Amyloid plaques are formed from amyloid β peptides (Aβ), which are cleaved by β- and γ-secretases from the amyloid precursor protein (APP). Mutations in the genes of APP and presenilin 1 and 2 (PS1, PS2), components of the γ-secretase complex, are linked to familial early onset AD (FAD) forms and have been shown to alter APP processing by enhancing the formation of Aβ_x-42_ peptide (Aβ42) [1].

Expression of human APP with single or double mutations in transgenic mouse lines leads to the formation of diffuse and neuritic plaques, which resemble the amyloid pathology seen in brain material from AD patients [2]. The onset and severity of this phenotype is further accelerated by crossing to mutant PS1 or PS2 transgenic mice [3]. These transgenic AD models have been proven to be a valuable tool for evaluating the effects of potential therapeutic agents, particularly targeting the amyloid pathology. For instance, active and passive immunization approaches against Aβ have been developed and successfully validated in animal models [1]. However, the use of many transgenic lines is limited by low breeding efficiencies, high premature death, late onset and high inter-animal variability of the pathology creating a need for improved animal models addressing these questions [3], [4].

An important consideration in the characterization of AD transgenic mouse models is the qualitative and quantitative evaluation of amyloid load in the brain. The total amount of Aβ in the brain is mainly quantified from total brain extracts by ELISA or Western Blot analyses whereas plaque morphology is currently best investigated in situ by immunohistochemistry or fluorescence staining [5], [6]. However, the plaque load, the number and size distribution of plaques are very important parameters which are not assessed by total Aβ levels but may be altered upon therapeutic intervention. Because conventional manual methods of plaque counting are very time-consuming and prone to errors, rapid and robust methods of quantitative plaque evaluation are required.

By neuron-specific over-expression of APP with the swedish double mutation (APP_swe_, K670N + M671L) and PS1 carrying the M146V mutation we have generated a double transgenic mouse line, ARTE10, which can be maintained homozygous for both transgenes. In order to characterize the amyloid pathology of the model, we established a semi-automated image analysis procedure that specifically and reproducibly quantifies plaque loads, plaque sizes and numbers in stained histological sections. Applying this novel method, we here demonstrate that ARTE10 mice consistently develop an early-onset and rapidly progressive AD-like cerebral β-amyloidosis with low inter-animal variability. Based on the phenotypic characteristics of the mouse line we suggest that ARTE10 is a mouse model well suited for studying amyloid-lowering therapies and presumably also for validation of new target candidates.

## Materials and Methods

### Animals

All experiments were done in accordance with the European Communities Council Directive of 24 November 1986 (86/609/EEC) and with the approval of the local institutional animal care committees (Landesamt für Natur, Umwelt und Verbraucherschutz, North Rhine-Westphalia, Regierung von Oberbayern, Munich, Amt für Gesundheit und Verbraucherschutz, Hamburg, Germany; Kantonales Veterinäramt Zurich, Switzerland). All efforts were made to minimize the number of animals used and their suffering.

### Generation of Transgenic Mice

To generate both transgenic constructs, the human APPswe and the human PS1M146V cDNAs respectively were cloned into the XhoI site of an expression cassette containing the murine Thy-1.2 gene [7]. The insert of both constructs were removed from the Vector backbone sequences by Not I digestion and gel purified prior to pronucleus injection. B6;CB-Tg(Thy1-PSEN1*M146V/Thy1-APP*swe)10Arte (ARTE10) mice were produced by pronuclear co-injection of Thy1-APPswe and Thy1-PS1M146V constructs into B6CBF1 eggs according to standard procedures [8].

### Quantitative Genotyping

The relative quantity of the human APPswe and the human PS1M146V transgenes was determined using the LightCycler technology (Roche Applied Science). First, standard curves were generated to determine the efficiency of PCRs with specific primers for APP: 5′-AAGATGCAGCAGAACGGCT-3′ and, 3′-CAGATCTAGCTCCAGGAAGGAGA-5′, and for PS1: 5′-GAGCCCTCGAAATTCGTTGA-3′ and 3′-TATCTCTTGCCGTCCTCGTGT-5. PCR amplification (95°C and 1 sec, 56°C and 5 sec, and 72°C and 5 sec) was performed in a volume of 20 µl containing LightCycler-FastStart DNA Master SYBR Green I mix (Roche Applied Science), 0.5 µM primers, 2 µl of a genomic DNA (gDNA) dilution series of a hemizygous ARTE10 mouse and additional 3 mM MgCl_2_. Melting curve analyses of the PCR products revealed single peaks at 82°C for APP, and 86°C for PS1 respectively, with no visible primer dimers. Quality and size of the qPCR product were determined applying the DNA 500 LabChip system using the 2100 Bioanalyzer (Agilent Technologies). Single peaks at the expected size of 85 bp for APP, and 146 bp for PS1 were observed in the electropherograms of the samples respectively. In an analogous manner, the qPCR protocol was applied to determine the PCR efficiency of murine APP gene intron, using the specific primers 5′-CCACAGGAGTTGCGGGAAT-3′ and 5′-AGTGTCTATCTACGGACCGAGT-3′. Melting curve analysis revealed a single peak at approximately 85°C with no visible primer dimers. Bioanalyzer analysis of the PCR product showed one single peak of the expected size (120 bp). For calculation of the standard values, first the logarithm of the used gDNA concentration was plotted against the threshold cycle value Ct for APP, PS1, and a murine APP gene intron, respectively. The slopes and the intercepts of the standard curves (i.e. linear regressions) were calculated.

In a second step, DNA amounts from controls and ARTE10 mouse gDNA samples were analyzed in parallel. The Ct values were measured and converted to ng gDNA using the corresponding standard curves: 10 ^∧^ (Ct value - intercept)/slope [ng gDNA]. Calculated gDNA concentration values of APP, and PS1, each were normalized to murine APP gene intron that was analyzed in parallel for each tested DNA probe, thus resulting values are defined as relative DNA levels.

### Antibodies

The following antibodies were used for immunofluorescence or -histochemistry: mouse anti-Aβ clone 4G8 (Signet), mouse anti-Aβ clone 6E10 (Signet), rabbit anti-Aβ (x-40) C-terminus-specific polyclonal 44–348 (Biosource), rabbit anti-Aβ (x-40) AB2050P (Araclon Biotech), rabbit anti-Aβ (x-42) C-terminus-specific polyclonal 44–344 (Biosource), mouse anti-Aβ (x-42) G2-13 (The Genetics Company), rabbit anti-Aβ polyclonal AB1 (Araclon Biotech), rabbit anti-oligomer conformation-specific polyclonal A11 (Invitrogen), mouse anti-APP near N-terminus (human-specific) clone LN27 (Zymed/Invitrogen), rabbit anti-APP near C-terminus (human, mouse) polyclonal AB5352 (Chemicon), mouse anti-tau (pS202) clone AT8 (Pierce Endogen), mouse anti-ubiquitin clone Ubi-1 (Zymed/Invitrogen), goat anti-AIF1 (anti-Iba1) polyclonal ab5076 (Abcam), rabbit anti-GFAP polyclonal AB5804 (Chemicon). Fluorophore-coupled donkey-anti-mouse, anti-rabbit or anti-goat antibodies were used as secondary antibodies. Antibodies were coupled to either Alexa488 (Molecular Probes), Cy3 or Cy5 (both Jackson ImmunoResearch).

### Immuno- and Amyloid-Staining

5 µm-thick paraffin sections from mouse brain hemispheres were used for all immuno- and amyloid stainings. Sections were stained with 1% Thioflavin S solution for 30 min at RT. Congo red amyloid staining was performed according to the method described by Puchtler et al. [9] by immersion in an alkalinized NaCl-saturated 80%-ethanolic solution of 0.2% Congo red for 15 min. Epitope retrieval by pretreatment with 70% formic acid (100264, Merck) for 15 min at RT was required in order to enhance the immunoreactivity of any anti-Aβ antibody used here; this formic acid pretreatment had no major influence (besides some slight signal enhancement) on the specific immunoreactivities of the antibodies used for the detection of ubiquitin (Ubi-1), abnormally phosphorylated tau (AT8), activated microglia (AIF1/Iba1) or reactive astrocytes (GFAP). Alternatively, heat-induced epitope retrieval in a pressure cooker with pH 6.0 sodium citrate buffer for 15 min was used in order to enhance the specific immunoreactivities of antibodies LN27 and AB5352. Immunoperoxidase staining was done using the HistoMouse-MAX kit (Zymed/Invitrogen) according to the manufacturer's instruction with the following modification: HistoGreen (Linaris) was used as chromogenic substrate and nuclear fast red (NFR, N-8002, 0.1% in aqueous solution containing 5% aluminium sulphate, Fluka/Sigma) was employed as counterstain. Stained sections were dehydrated through ascending ethanol (70%, 96%, absolute), cleared in two changes of xylene and mounted with Histomount (Zymed/Invitrogen).

For immunofluorescence stainings, sections were blocked with 3% bovine serum albumin (BSA, Fluka/Sigma) in PBS for 15 min and probed with one, two or three simultaneous primary antibodies diluted in 1% BSA/PBS over night at 4°C, washed with PBS, blocked again with 3% BSA/PBS and incubated with one, two or three simultaneous fluorophore-conjugated secondary antibodies, washed, and coverslip-mounted with ProLong Gold Antifade mounting medium (Invitrogen/Molecular Probes). Optionally, nuclei were stained by adding 0.5 µM DAPI (32670, Fluka/Sigma) before the last washing steps. HistoGreen/NFR-stained sections were scanned with the Minolta Dimage Scan Elite 5400 slide scanner at highest resolution (5400 dpi, corresponding to approximately 5 µm per pixel) in the colour positive mode, for which purpose the slide scanning rack had been adapted to hold 3 standard microscope slides. Fluorescence or brightfield microscopy was performed with an Olympus BX51 or Zeiss AxioImager Z.1 microscope. Digital micrographs were acquired with a ColorView II charge-coupled display (CCD) camera (Soft Imaging System, Olympus) or AxioCam MRm Rev. 3.0 (Carl Zeiss). Entire-view micrographs of parasagittal forebrain sections were recorded through a 2x objective followed by a 0.5x TV adaptor.

### Image Analysis and Plaque Quantification

Digital images were evaluated with the Acapella™ data analysis software (Evotec Technologies, Fig. S2). An object-based algorithm was developed for the specific and sensitive recognition of individual plaques and for the quantitative assessment of the relative plaque load, calculated as the percentage of the area covered by plaques over the total area of interest (i.e. the sum of pixels representing plaque signals divided by the sum of all pixels representing the region of interest), the number of individual plaques per area and their size distribution. Regions of interest (namely, the neocortex and the hippocampus) were defined by manual segmentation in accordance with the anatomical delineations given by Paxinos and Franklin (1997) [10] using a state-of-the-art image editing software (Adobe PhotoShop CS). The resulting region-of-interest images were converted from RGB into HSV format and split into the three corresponding 8-bit channels respectively representing the hue, the saturation and the intensity value of each pixel. The combination of different parameters from two different channels ensured both high sensitivity and specificity of plaque recognition. Detection of HistoGreen-stained plaques was most sensitive and robust in the hue channel, and the specificity of separation from the pink-red counterstained tissue background was also optimal in the hue channel. The saturation channel, however, was best suited for discriminating and eliminating contaminations or otherwise interfering signals e.g. resulting from dust particles, small irregularities or folds in the tissue section, hence further enhancing the specificity of plaque recognition. A three-step algorithm was designed. In a first step, individual global thresholds were dynamically generated and applied to each channel in order to create masks which were subsequently combined through an adequate set of operations in order to find a good overall approximation of plaque locations. As a side effect of ensuring maximum sensitivity of detection, the resulting preliminary plaques were generally somewhat overestimated in size, and clusters of closely neighbored individual plaques were sometimes coalesced to simulate one or more giant plaques. To correct for this error, in a second step, the topology of individual and between-plaque borders was determined by reducing the preliminary plaques to few extremal points, typically approximating individual or local barycenters, and by using these points as seeds for a conventional watershed algorithm. Growth of watershed basins was restricted to the preliminary plaque contours. In a third step, each object having passed step two was checked for its plaque-likeness on a rough morphometrical plane, and objects that were too small or extremely skewed were presumed to be unspecific and therefore removed. All other objects were recognized as plaques, and the plaque size (pixel count) was recorded for quantitative analyses specified above. A lower limit for meaningful plaque detection was determined by direct microscopic evaluation of the sections with the lowest plaque loads. The visually confirmed threshold was set at 0.005% (plaque area per area of interest). At least six standardized parasagittal sections containing major portions of the neocortex and hippocampus were analyzed per animal and mean values were generated for each individual.

### Differential Extraction of Aβ x-40 and Aβ x-42 Peptides

Frozen brain hemispheres were homogenized in 19 volumes of tris buffer (150 mM NaCl, 20 mM Tris pH 8.0+ Complete, Roche Applied Science) using a Dounce glass homogenizer with a Teflon pestle and centrifuged at 53000 rpm (TLA-120.2 rotor, Beckmann) for 30 min at 4°C. Pellets were resuspended in the same volume of 70% formic acid, kept on ice for 30 min and centrifuged likewise. Supernatants were neutralized with 19 volumes of 1 M Tris pH 11.3. Aβ peptides were quantified in supernatants of both extractions using commercially available ELISA kits (hAmyloid β40 ELISA TK40HS, hAmyloid β42 ELISA TK42HS, The Genetics Company) recognizing human but not endogenous murine Aβ. Total protein was measured using a protein detection kit (BCA test, Pierce) and results are expressed as pg Aβ per µg protein.

### Substance Application

The γ-secretase inhibitor MRK-560/compound 32 [11] was used. 3 to 7 months old hemizygous ARTE10 mice received either vehicle (10% ethanol in corn oil), 1, 10, 30 or 100 mg/kg MRK-560 suspended in vehicle solution by oral gavage at 10 ml/kg. After 4 h brains were excised and the amount of soluble Aβ was determined as described above using the brain lysis buffer from the ELISA kit (The Genetics Company).

### Ex Vivo Autoradiography

A 17 months old hemizygous female ARTE10 mouse (29.7 g) received about 100 µl of a solution containing 0.46±0.02 MBq of N-[^3^H-methyl]-6-OH-BTA-1[12] in PBS through one of its lateral tail veins. The animal was guillotined at 40 min post-injection, the full brain was removed, rapidly frozen in dry ice and stored for three days in air-tight vials at −75°C until sectioning. For tissue analyses, the brain was cut into 10 µm coronal sections on a cryostat (Leica Microsystems). The frozen sections were thaw-mounted on dilute poly-L-lysine coated (mol wt >300.000, (1∶50) 0.01%w/v in water, Fluka/Sigma) microscopy slides, dried in ambient air and stored at −75°C. The deep frozen CNS sections were dried at ambient conditions for 30 min and subsequently covered with scintillation foil. Digital autoradiography images were acquired with the M40 series of μ-Imager™ (Biospace lab, France). The resolution with tritium is 20 µm, the detection threshold for tritium is 0.4 cpm/mm^2^ and the smallest pixel size is 1 µm. Data acquisition was controlled with μ-Acquisition software. The data was exported with β-Vision+ software (both from Biospace Lab, France) for processing in Adobe Photoshop CS3 Extended (Version 10.0.1). The color look-up table used here (Orange Hot) was imported from NIH ImageJ (Version 1.43 g).

### qPCR Expression Analysis

#### Brain tissue dissection

Brain tissues from ARTE10 mice and control subjects, were collected. Sample sections from each tissue were fixed in paraformaldehyde. Brain areas for mRNA expression analysis were stored at -80°C until RNA extractions were performed.

#### Isolation of total mRNA

Total RNA was extracted from frozen brain tissue by using the RNeasy kit (Qiagen) according to the manufacturer's protocol. The accurate RNA concentration and the RNA quality were determined applying the Eukaryote total RNA Nano LabChip system by using the 2100 Bioanalyzer (Agilent Technologies). For additional quality testing of the prepared RNA, i.e. exclusion of partial degradation and testing for DNA contamination, specifically designed intronic GAPDH oligonucleotides and genomic DNA as reference control were utilized to generate a melting curve with the LightCycler technology (Roche Applied Science) as described in the supplied protocol by the manufacturer.

#### cDNA synthesis

Here, total RNA was used as starting material, which had been extracted as described above. For production of cDNAs, the cDNA Synthesis System was performed according to the manufacturer's protocol (Roche Applied Science). The accurate cDNA concentration was determined by photometric analysis (OD 260/280 nm).

#### Quantitative RT-PCR

mRNA expression analyses of the genes coding for Synaptophysin (Syp), Disk large homolog 4 (Dlgh4), and Drebrin (Dbn1) were performed using the LightCycler technology (Roche Applied Science). The relative quantity was determined as following. First, standard curves were generated to determine the efficiency of PCRs with specific primers for Syp: 5′-TCCCTCTGCCCCTCCTAACT-3′ and, 3′-GTTCTCATCTCCCCACCTCCAC-5′, for Dlgh4: 5′-GCACCGACAACCCACACAT-3′ and 3′-CAGTTACTTCACCTACAGGCC-5′, and for Dbn1: 5′-GGAGATGAAGCGGATTAACCG-3′ and 3′-TTCTTCCGAGACCTGCGGT-5′.

PCR amplification (95°C and 1 sec, 56°C and 5 sec, and 72°C and 5 sec) was performed in a volume of 20 µl containing LightCycler-FastStart DNA Master SYBR Green I mix (contains FastStart Taq DNA polymerase, reaction buffer, dNTP mix with dUTP instead of dTTP, SYBR Green I dye, and 1 mM MgCl_2_; Roche Applied Science), 0.5 µM primers, 2 µl of a cDNA dilution series from mouse total brain and additional 3 mM MgCl_2_ (for Dbn1 1 mM MgCl_2_). Melting curve analyses of the PCR products revealed single peaks at 84°C for Syp, 87.5°C for Dlgh4, and 86.5°C for Dbn1 respectively, with no visible primer dimers. Quality and size of the qPCR product were determined applying the DNA 500 LabChip system using the 2100 Bioanalyzer (Agilent Technologies). Single peaks at the expected size of 103 bp for Syp, 134 bp for Dlgh4, and 98 bp for Dbn1 were observed in the electropherograms of the samples respectively. In an analogous manner, the qPCR protocol was applied to determine the PCR efficiency of Cyclophilin B (Ppib), using the specific primers 5′-GATGTCATCATTGTCGACTCCG-3′ and 5′-GCTCTCTAC TCCTTGGCAATGG-3′. Melting curve analysis revealed a single peak at approximately 83.5°C with no visible primer dimers. Bioanalyzer analysis of the PCR product showed one single peak of the expected size (71 bp). For calculation of the standard values, first the logarithm of the used cDNA concentration was plotted against the threshold cycle value Ct for Syp, Dlgh4, Dbn1, and Ppib, respectively. The slopes and the intercepts of the standard curves (i.e. linear regressions) were calculated.

In a second step, mRNA expression from controls and ARTE10 mouse brain samples were analyzed in parallel. The Ct values were measured and converted to ng total brain cDNA using the corresponding standard curves: 10 ^∧^ (Ct value - intercept)/slope [ng total brain cDNA]. Calculated cDNA concentration values of Syp, Dlgh4, and Dbn1, each were normalized to Ppib that was analyzed in parallel for each tested tissue probe, thus resulting values are defined as arbitrary relative expression levels.

### Behavioral Analyses

Three cohorts of hemizygous and homozygous transgenic mice as well as wild type litter mates were analyzed. One cohort was repeatedly tested in a longitudinal study design at 4, 8 and 12 months of age. The other cohorts were analyzed in a cross-sectional study at 8 and 12 months respectively. Number for each group were: longitudinal cohort, 15 wild type (5 females+10 males), 16 hemizygous (8 females + 8 males), 13 homozygous (9 females + 4 males, 2 females died before the age of 12 months); cross-sectional cohort at 8 months, 14 wild type (5 females+9 males), 15 hemizygous (5 females + 10 males), 12 homozygous (5 females + 7 males); cross-sectional cohort at 12 months, 14 wild type (9 females + 5 males), 14 hemizygous (9 females + 5 males), 14 homozygous (8 females + 6 males). All mice were age-matched and were tested in an identical test battery.

The Morris water maze test was performed in a circular swim tank (diameter 150 cm, height 50 cm), the quadratic target platform (14×14 cm) was made of metallic wire mesh and was hidden 0.5 cm below the water surface in the center of one of four pool quadrants (target quadrant). A circular zone of 53 cm in diameter around the target platform was defined as target zone (12.5% of the total area of the pool). Movement of the mice was tracked by a video camera and EthoVision© Software (Noldus, Netherlands), data analysis was done with Wintrack Software [13]. Each mouse was trained for a total of 18 training trials to find the position of the hidden platform which remained fixed for each animal throughout all trials of the task. A probe trial was conducted on day 4 in which the platform was removed from the pool and the path that each mouse swam was recorded over a 60 sec period. In the longitudinal study the position of the escape platform for each animal was changed at each of the three time points.

The object recognition test was performed in the open field box (50×50×50 cm). After a habituation session animals were placed for 10 min into the box with two identical objects. A recall session was conducted 24 h later in which one object was replaced by a new object. Activity was recorded and analyzed using a video camera and EthoVision© Software (Noldus, Netherlands) while exploration was scored manually. Exploration was defined as approach to an object with the animal's head at a minimum distance of 1 cm. Preference for the new versus the old object was calculated according to the following formula: preference  =  exploration time new object/total exploration time.

### Statistical Analyses

Unless otherwise noted, statistical analyses of means + SEM were done using StatView© statistical software (SAS Institute). In case of normally distributed data One-Way ANOVA for genotype or dosage was performed for single measures at each time point, repeated measures ANOVA for several data points for each animal within one test. Fisher's PLSD was used for post-hoc analysis. Use of unpaired t-test was indicated where appropriate. One-sample t-tests were performed to test significance in comparison to chance level. Regression diagnostic was done using S-Plus 8.0.4 for Linux. Non-parametric Mann-Whitney test (two-tailed, 95% confidence level) was performed using GraphPad Prism 4 (GraphPad Software, Inc).

## Results

### Generation of Transgenic Mice

Several independent transgenic founder lines were established by co-injection of human APP_swe_ and PS1_M146V_ each driven by the Thy-1 promoter. In all tested lines, the two transgenes were co-inherited without segregation over several generations. Expression of the transgenes was analyzed by Northern blot analysis and independent transgenic lines were initially screened for formation of amyloid plaques in the brain by congo red staining (data not shown). Based on high expression level and early plaque formation line ARTE10-729 was selected for propagation and subsequent analysis. Breeding performance of this line was indistinguishable from C57BL/6 mice, even when retained as a homozygous line (C57Bl/6: 3.0±0.2 pups per female per month, hemizygous ARTE10: 3.5±0.4 pups per female per month, homozygous ARTE10: 3.1±0.4 pups per female per month). Also, the rate of premature death was quite low in this line. During the longitudinal study all hemizygous ARTE10 mice reached the age of 12 months (100% survival) and survival was also 100% in the wild type littermate group, whereas only 2 out of 13 homozygous ARTE10 mice died before the age of 12 months (85% survival).

### Assessment of Transgene Expression in ARTE10 Brain

**Figure 1:**
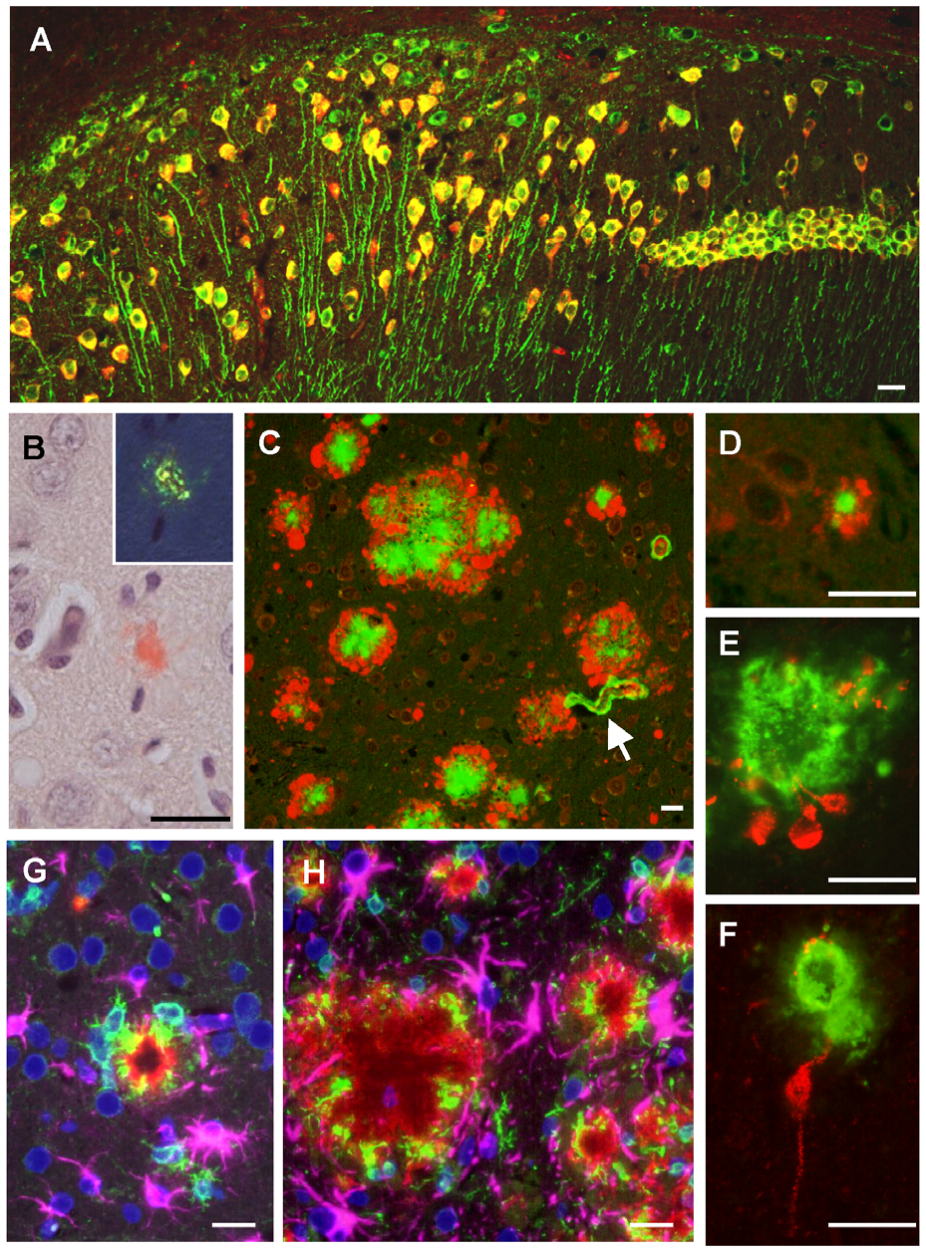
Amyloid pathology and glial inflammation in ARTE10 mouse brain. **A**, Double-immunofluorescent colocalization of the transgenic human APP (*red*, human-specific antibody against the N-terminal ectodomain) with the neuron-specific somato-dendritic marker MAP2 (*green*) in the subiculum and the CA1 region of the hippocampus. The transgene is amply expressed in most (>80%) neurons (*yellow* merge colour), whereas non-neuronal cells show no signals beyond background. **B**, Congo-red stained plaque core (*red*) in the frontal cortex of a hemizygous ARTE10 mouse at 5 months of age. The green bi-refringence in polarized light (inset) is diagnostic of amyloid. **C, D**, Thioflavin-S positive plaque cores (*green*), each encircled by a sphere (corona) of dilated, strongly ubiquitin-positive dystrophic neurites (*red*) in the subiculum of a homozygous ARTE10 mouse at 3 months of age (D) and in the frontal cortex of a 19 months old hemizygous mouse (C), the latter with concomitant amyloid angiopathy (arrow). **E, F**, AT8-positive hyperphosphorylated tau (*red*) in dystrophic neurites adjacent to plaque cores (*green*: Aβ40 and Aβ42) in homozygous ARTE10 mice at 12 (E) and 8 (F) months of age. **G, H**, Triple-immunofluorescent demonstration of plaque-associated mixed glial inflammation: Activated microglia (*green*, AIF1/Iba1) and reactive astroglia (*magenta*, GFAP) colonizing the periphery of amyloid cores (*red*: Aβ/6E10) in the subicula of a homozygous 5 months (G) and a hemizygous 13 months (H) old ARTE10 animal (*blue*: nuclei/DAPI). Scale bar, 20 µm.

Transgene expression was analyzed by double-immunofluorescent labeling of human APP and MAP2, a specific somato-dendritic marker of all CNS neurons, on brain sections of ARTE10 mice. The transgenic human protein was abundantly and selectively expressed by the vast majority (>80%) of neurons in the hippocampus (Fig. 1a) and by approximately 50% of neurons in the neocortex (data not shown). No transgenic protein was detectable in non-neuronal cells in the brain, particularly glial and vascular smooth muscle or endothelial cells, confirming neuron-specific transgene expression (Fig. S1).

### AD-Like Aβ-Related Neuropathology, Morphology of and Composition of β-Amyloid Plaques

ARTE10 mice developed cerebral β-amyloidosis with similar morphology and composition as in human AD-affected brain, including mainly dense-core, and to a lesser extent diffuse plaques as well as amyloid angiopathy. The plaques were composed of Aβ peptide (both Aβ40 and Aβ42 forms, Fig. 1e–h and Fig. S3), and their amyloid nature was evidenced by staining with congo red, imparting the characteristic green bi-refringence in polarized light (Fig. 1b), or with thioflavin S (Fig. 1c–d, Fig. S4). Diameters of the congophilic plaque cores ranged from 5 to 200 µm, averaging between 20 and 50 µm.

The dense-core plaques were each encompassed by a sphere (corona) of early-dystrophic, swollen neurites featuring pronounced accumulation of ubiquitin (Fig. 1c–d, Fig. S4) and synaptophysin (Fig. S5). In ageing homozygous ARTE10 mice, a minority of dystrophic neurites contained AT8-positive material, indicating nascent tau hyperphosphorylation (Fig.1e–f). However, argyrophilic neurites, tangles or neuropil threads were not observed even in animals aged up to 20 months.

Diffuse plaques, i.e. accumulations of Aβ peptide lacking a congophilic core, were only observed in the presence of very high total plaque loads, forming small satellite deposits in proximity to dense-core plaques. Like in human brain, diffuse deposits were composed almost exclusively of Aβ_x-42_ species (Fig. S3). However, diffuse deposits were absent from ARTE10 brains with low plaque burden or with beginning plaque deposition, where even the earliest and smallest detectable Aβ deposits already consisted of mature, congophilic amyloid (Fig. 1b+d, Fig. S4).

All amyloid plaques were accompanied by marked mixed glial inflammation with many activated microglia and reactive astrocytes populating the outer plaque spheres (Fig. 1g–h, Fig. S4). Additional cerebral amyloid angiopathy of intracortical, leptomeningeal and choroidal blood vessels regularly occurred in ARTE10 mice with advanced plaque deposition (arrow in Fig. 1c).

Intracellular Aβ was detected in several pyramidal neurons of the cortex and hippocampus. This intraneuronal Aβ immunoreactivity was found already in young ARTE10 mice. It exhibited a speckled pattern within the perikarya. Interestingly, some neurons showed a similar pattern of intracytoplasmic immunoreactivity with the anti-oligomer conformation-specific antibody A11 (Fig. S6).

### Pattern and Sequence of Plaque Deposition

**Figure 2:**
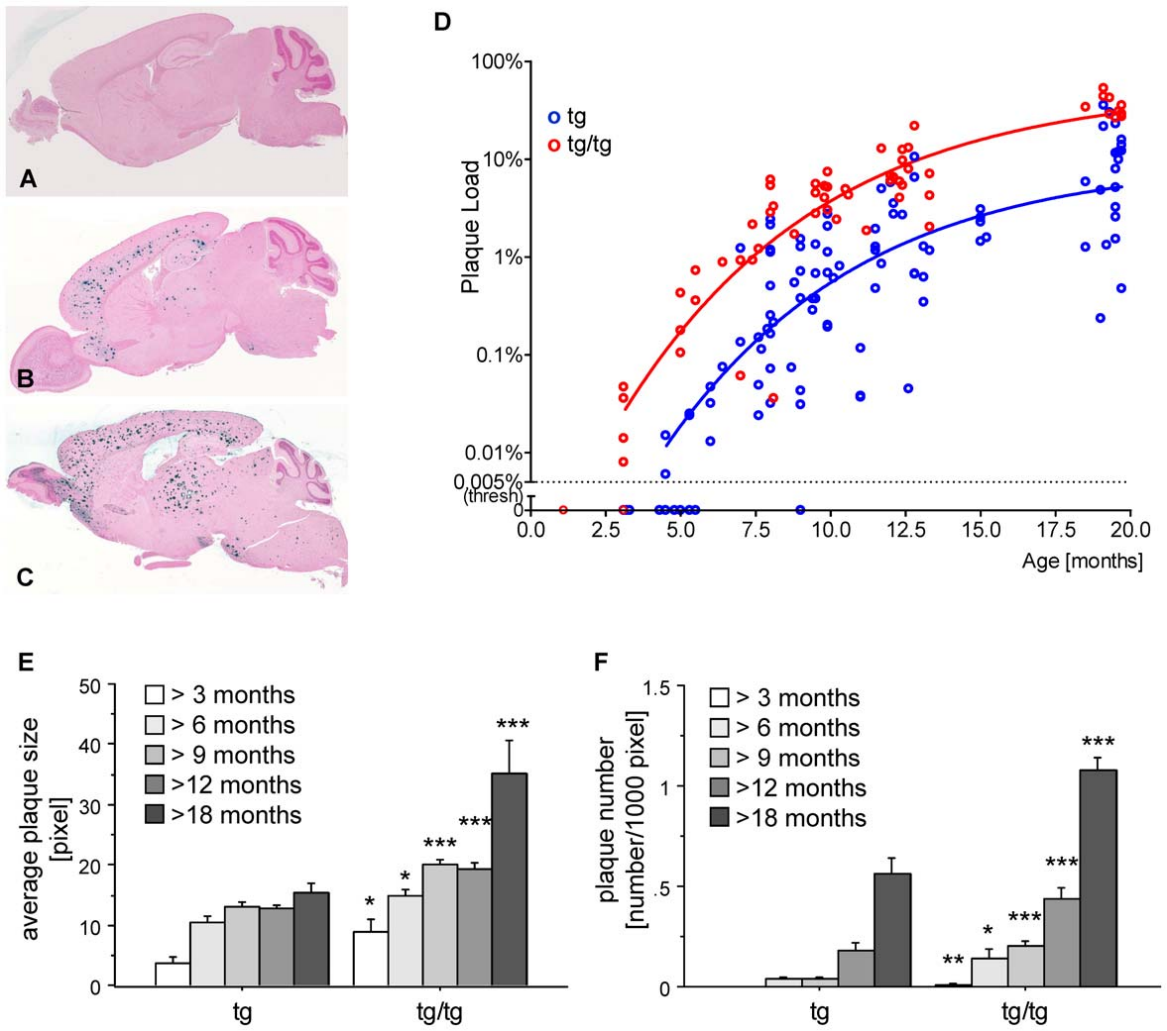
Onset and development of plaques in ARTE10 mice. **A–C**, Digital micrographs of parasagittal forebrain sections from homozygous transgenic mice immunohistochemically stained for β-amyloid plaques (*dark blue-green*) at 3 (A), 8 (B), and 13 months of age (C). **D–F** Plaques are quantified using Acapella™ plaque recognition software in regions of interest on stained mouse brain sections. **D**, Plaque load is expressed as percentage of plaque area per total area of neocortex and hippocampus across ages in homozygous (*red*) versus hemizygous mice (*blue*). Each symbol represents one animal. The average size (E) and number of plaques per area (F) progressively increase with age in hemizygous (tg) and homozygous (tg/tg) ARTE10 mice. Data are expressed as means + SEM; * p<0.05, ** p<0.01, *** p<0.0005, tg versus tg/tg at the respective age (unpaired t-test).

The spatio-temporal development of cerebral amyloid plaque pathology was monitored by histological examination of hemizygous and homozygous ARTE10 mouse brains at different ages (Fig. 2a–c, Fig. S7+S8).

The first plaques always occurred in the anterior neocortex and in the subiculum as early as 3 and 5 months after birth in homozygous and hemizygous animals, respectively, shortly followed by the posterior neocortex, the CA1-4 regions of the hippocampus, the amygdala and related limbic structures, and the thalamus. Brain regions that were affected later include the olfactory bulb, the colliculi, the brainstem, and the striatum, where mainly ventral parts of the putamen developed plaques. At advanced stages, i.e. from 8 months onward in homozygous followed by hemizygous mice 2–3 months later, the largest plaques were regularly observed in the thalamus. These giant plaque cores were sometimes subject to microcalcification, a phenomenon that was never observed in any other brain region. The regions that were affected first consistently maintained their lead over the regions that became affected later, i.e. the anterior neocortex and the subiculum always showed the highest plaque densities. Plaques were never observed in the cerebellum.

### Quantification of Plaque Load

In order to quantitatively evaluate plaques visualized immunohistochemically in brain sections, a computerized image analysis and object recognition algorithm was designed (Fig. S2).

We analyzed the neocortex and the hippocampus from a total of 176 hemizygous or homozygous ARTE10 mice of different ages ranging from 3 to 20 months. The plaque load progressively increased with age, exhibiting saturation kinetics (Fig. 2d). The onset, rate and maximum levels of plaque deposition were transgene dose-related, i.e. starting earlier, increasing faster and peaking higher in the homozygous as compared to the hemizygous condition. The same correlation was revealed for the number and average size of plaques (Fig. 2e, f). 100% penetrance of the plaque phenotype could be observed by 5 and 10 months of age in homozygous and hemizygous mice, respectively. Aged ARTE10 mice of 19–20 months reached plaque loads of 10.5% (+/− 2.2%, range 0.2–35.6%) in hemizygous mice and 35.2% (+/− 2.8%, range 26.4–53.2%) in homozygous animals.

### Comparison of Plaque Load to Aβ Levels in Brain Homogenates

**Figure 3:**
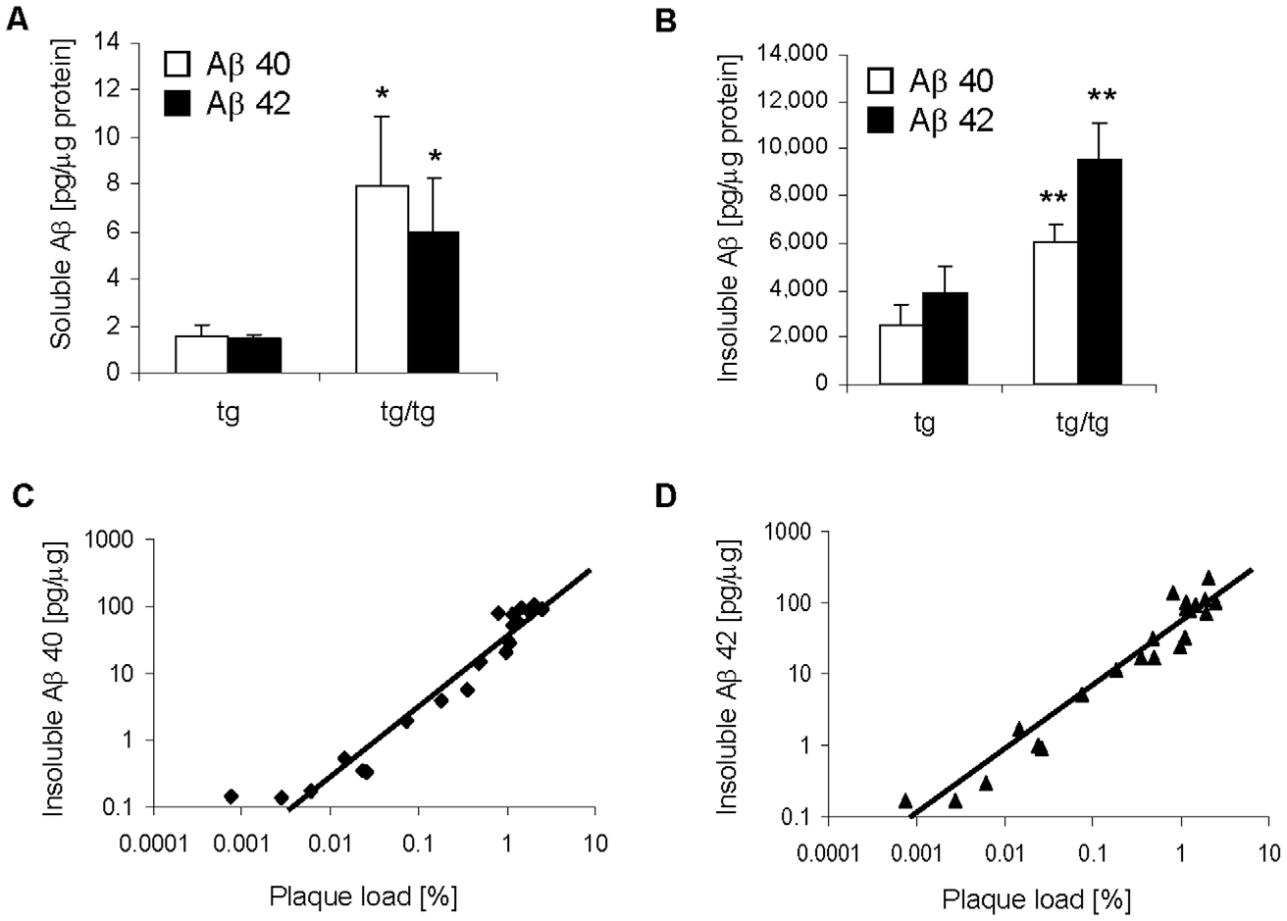
Correlation between insoluble Aβ and plaque burden. **A, B**, Quantification of Aβ peptides in brain extracts from hemizygous and homozygous ARTE10 mice. 12 months old mice from the cross sectional study were used for the analysis. Soluble (A) and insoluble Aβ (B) was extracted with Tris buffer and formic acid, respectively, and the amount of Aβ40 (open bars) and Aβ42 (filled bars) was quantified. The brains of homozygous mice contained more soluble as well as insoluble Aβ in comparison to hemizygous mice. Data are expressed as means + SEM. Tg, hemizygous transgenic mice; tg/tg, homozygous transgenic mice. * p<0.05, ** p<0.01, tg versus tg/tg at the respective age (unpaired t-test). **C, D**, Correlation of insoluble Aβ and plaque load calculated by Acapella™ software. Insoluble Aβ40 (C) and Aβ42 (D) were extracted from one brain hemisphere of hemizygous ARTE10 mice of different age (4 to 15 months old). Plaque load was calculated by Acapella™ image analysis software from sections of the corresponding second hemisphere after staining against β-amyloid. A strong correlation between plaque burden and insoluble Aβ was found. C, R^2^ = 0.86; D, R^2^ = 0.69.

Brain homogenates of 12 months old ARTE10 mice were subjected to biochemical quantification of human Aβ40 and Aβ42. Differential extraction procedures were applied in order to determine the levels of either soluble or insoluble forms of Aβ species. More soluble and insoluble Aβ was detected in the brains of homozygous in comparison with hemizygous transgenic animals (Fig. 3a, b). The ratio of insoluble Aβ42 to Aβ40 was approximately 1.5 and comparable between homozygous and hemizygous mice (Fig. 3b). Hemizygous mice of different ages, ranging from 4 to 15 months, were used in order to mutually cross-validate the histology image-based plaque load quantification with biochemical analyses (Fig. 3c, d). Both hemispheres of each mouse brain were employed, one for histological plaque quantification, the other for biochemical measurement of insoluble Aβ. The results of the corresponding hemispheres per animal were compared directly. Levels of insoluble Aβ40 and Aβ42 strongly correlated with the histological plaque burden as determined by the image analysis confirming that both methods exhibit equivalent quantitative power (Fig. 3c, d).

### [^3^H]PIB Distribution in the Brain Parenchyma of ARTE10 Mice

**Figure 4:**
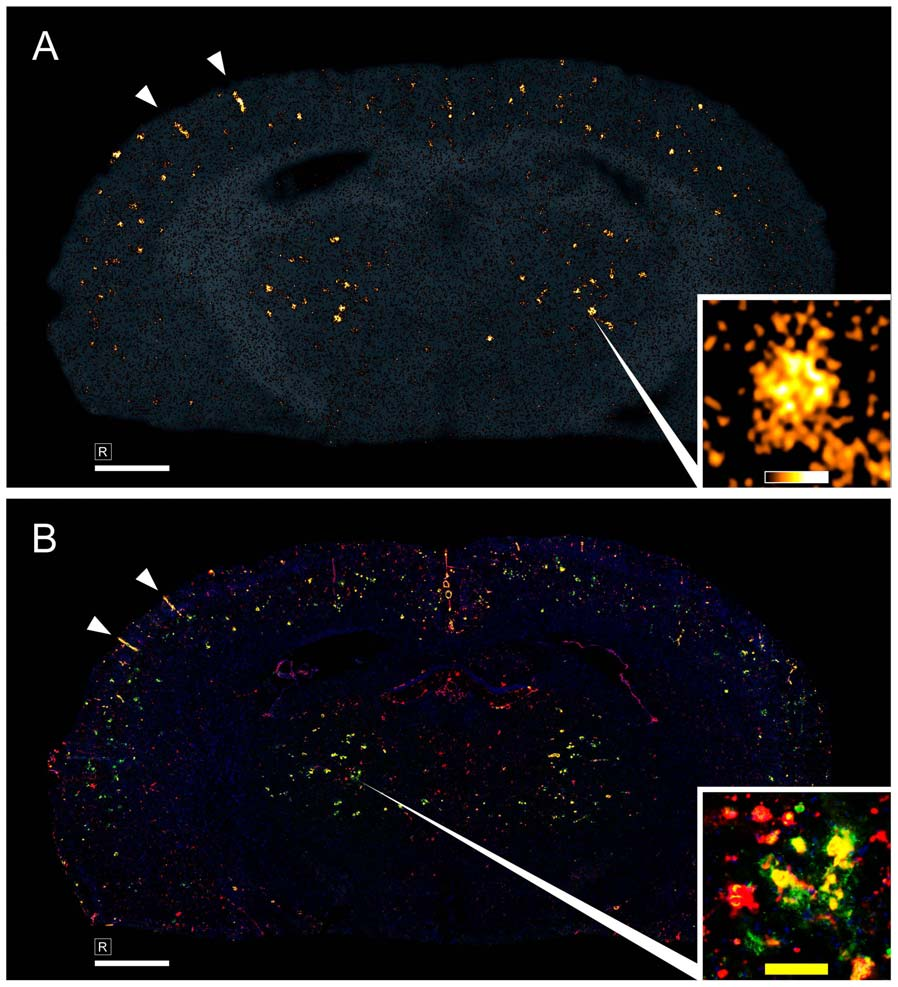
In vivo binding of [^3^H]PIB to amyloid plaques. **A**, Ex vivo digital autoradiography co-registered to its optical scan of a coronal brain section at about −0.94 Bregma of a 17 months old hemizygous female ARTE10 mouse killed 40 min after intravenous administration of [^3^H]PIB. The dotted pattern demonstrates uptake of tracer in Aβ aggregates of cortical and thalamic regions. **B**, Immunofluorescence stain for Aβ40 (*green*), Aβ42 (*red*) and nuclei (*blue*) of a close parallel section. *Insets:* single plaque taken from marked positions. *Arrowheads:* Tracer binding and Aβ staining of amyloid-β in vessels. *Scale bars in overview:* 1 mm. *Color and scale bars in insets:* 100 µm

In order to evaluate potential use of ARTE10 mice for in vivo analyses of amyloid plaques, [^3^H]PIB, a specific Aβ tracer, was administered intravenously followed by ex vivo digital autoradiography of brain sections. Amyloid-β plaques were shown as a dotted pattern of focal tracer retention in the whole of the cortex and most thalamic regions (Fig. 4a). The autoradiography method used here is sufficient to resolve finer structures beyond the level of plaque demarcation (Fig. 4, inlets). This picture correlated very well with the distribution pattern and appearance of Aβ40 and Aβ42 in the immune fluorescence staining of a parallel section close by (Fig. 4b). Also, amyloid in vessels can clearly be seen in both modalities (Fig. 4a and 4b, arrowheads). Hence, these data are consistent with the anticipation that [^3^H]PIB crossed the blood-brain barrier of ARTE10 mice and significantly bound to Aβ plaques in these animals in vivo.

### Expression Analysis of Pre- and Post-Synaptic Markers

**Figure 5:**
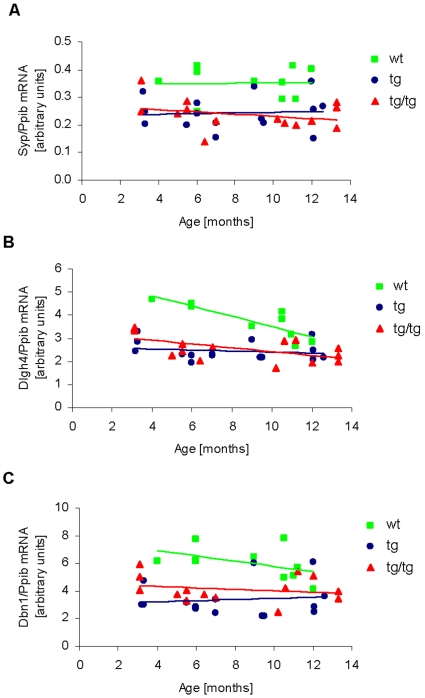
mRNA expression analysis of synaptic marker proteins in the brain of ARTE10 mice. **A**, Synaptophysin (Syp), **B**, Disk large homolog 4 (Dlgh4), and **C**, Drebrin (Dbn1) mRNA expression in hemizygous and homozygous ART10 mice were analyzed in comparison to wild type animals. Material from comparable brain regions of mice ranging from 3 to 13 months of age was used for each of these studies. mRNA has been extracted from the brain material and expression of Synaptophysin, Disk large homolog 4, and Drebrin was measured applying real-time quantitative PCR. For normalization, each ratio of Synaptophysin, Disk large homolog 4, and Drebrin values with Cyclophilin B (Ppib) have been calculated and are shown as linear regression lines. In comparison to wild type mice hemi- as well as homozygous mice revealed a significant lower mRNA level of Synaptophysin (p = 0.0011; 0.0003), Disk large homolog 4 (p = 0.0003; 0.0011), and Drebrin (p = 0.0003; 0.0002) respectively (see also Fig. S9); statistical analyses were done by Mann-Whitney test. Additionally, in wild type mice mRNA expression of Dlgh4 and Dbn1 were decreased with aging (p = 0.0013; 0.0253); no such decrease with aging was shown for Synaptophysin (p = 0.5140). Analysis was done by regression diagnostic.

Applying real-time quantitative PCR, the mRNA expression of the presynaptic marker protein Synaptophysin (Syp) as well as the postsynaptic markers Disk large homolog 4 (Dlgh4) and Drebrin (Dbn1) were analyzed in brain samples from ARTE10 mice (Fig. 5, Fig. S9). Gene expression revealed that ARTE10 mice expressed Syp mRNA at a level of approximately 70% that of wild type mice (i.e., a 30% reduction) and without any obvious difference between hemi- and homozygous mice (Fig. 5a, Fig. S9). The levels of Syp mRNA were shown to stay constant at all ages examined. Gene expression analyses of Dlgh4 (Fig. 5b) and Dbn1 (Fig. 5c) revealed a similar result of a decrease of approximately 30% compared to Syp at early age points (Fig. S9). In wild type mice mRNA expression levels of Dlgh4 and Dbn1 were decreased with age whereas expression of Syp mRNA stayed constant. In contrast, in transgenic mice mRNA expression of all three synaptic markers revealed no further decrease along with aging.

### Behavioral Analyses

**Figure 6:**
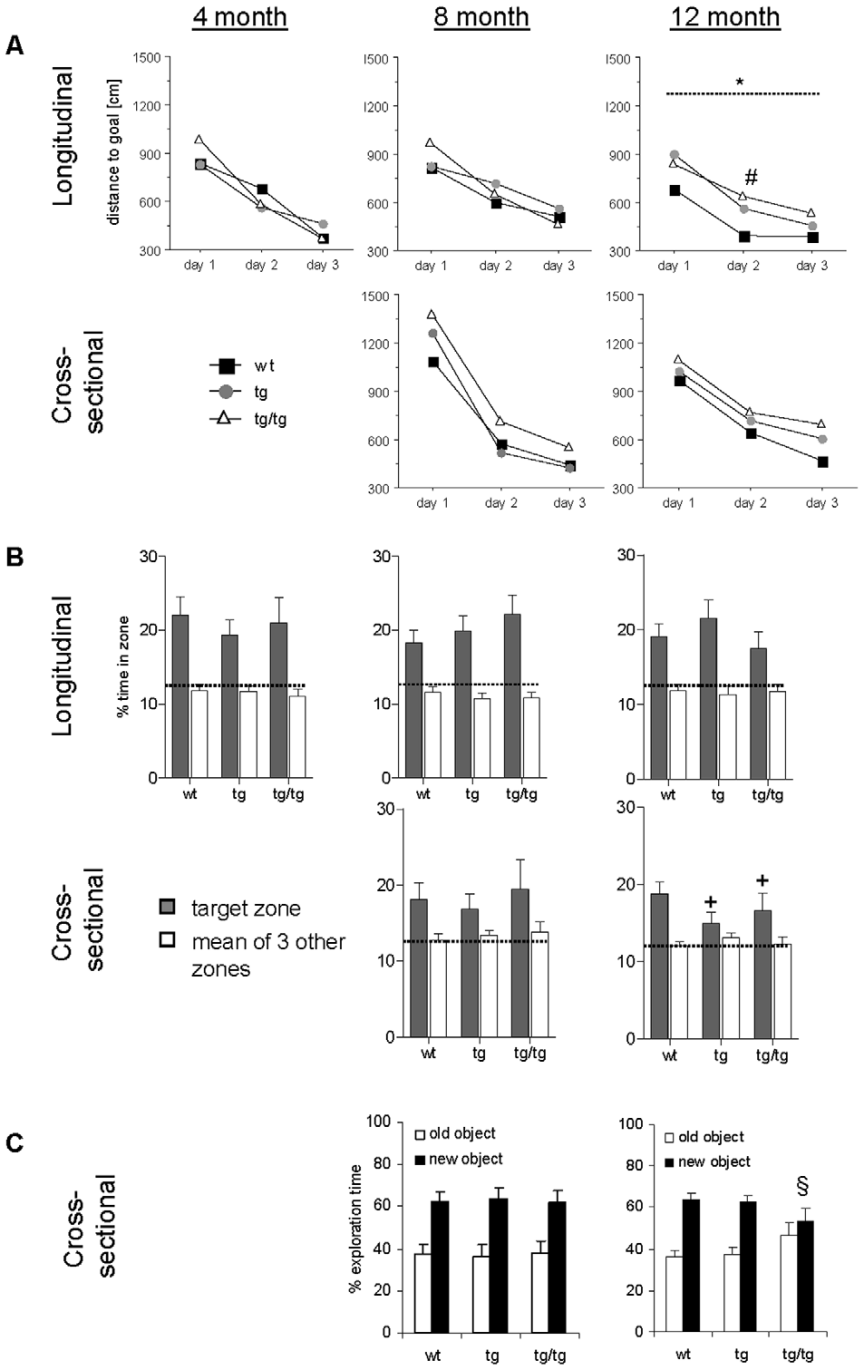
Learning and memory of ARTE10 mice in behavioral tests. **A**, In the water maze task 12 months old ARTE10 mice from the longitudinal cohort are impaired in spatial learning. Animals were trained to learn the position of a hidden escape platform in the water pool. In the longitudinal study animals were tested repeatedly every 4 months until they reach 12 months of age. In the cross sectional study design different testing groups with naïve animals were used at each time point. Each data point represents the mean of 6 trials per animal. (*) p = 0.0407 between groups over days (repeated measures ANOVA); (#) wt v/s tg/tg, p = 0.0154 (unpaired t-test). **B**, After completion of the water maze training memory for the platform position in the target zone was assessed during a probe trial in which the platform was removed from the pool. For each group the percentage time in target zone (filled bar) is shown in comparison to the mean percentage time for the other three zones (open bar). (+) ≤12.5%, tg, p = 0.052; tg/tg, p = 0.052 (one sample t-test). Data are expressed as means + SEM. Dotted lines represent chance levels (12.5%). **C**, Performance of mice in the object recognition test. Naïve homozygous ARTE10 mice display deficits in episodic memory at 12 months of age as demonstrated by lacking preference for a new over an old object. Preference for the new object (filled bar) is expressed as percentage exploration time in comparison to exploration of the old object (open bar). Data are expressed as means + SEM. ($)≤50%, tg/tg, p = 0.293 (one sample t-test); tg, hemizygous transgenic mice; tg/tg, homozygous transgenic mice; Wt, wild type litter mates.

In both a longitudinal and a cross-sectional design, three cohorts of mice were characterized in a battery of behavioral tests at different ages (Fig. 6; results of sensorimotor, locomotory and exploratory behavioral tests can be found in Supporting File S1 and Tables S1 and S2).

A place navigation protocol measuring the spatial learning and memory abilities of ARTE10 mice was performed in the Morris water maze. Since swim speeds differed between groups at various time points (data not shown), the animal's path-length to reach the platform were analyzed. At 4 and 8 months of age, mice of all groups learned consistently to locate the platform during training (Fig. 6a). By 12 months, a significant effect of genotype could be found in the longitudinal study design (repeated measures ANOVA, F_(2,39)_ = 3.48, p = 0.041; post-hoc analysis wt vs. tg, p = 0.037, wt vs. tg/tg, p = 0.024). Longer swim distances were mainly needed at day 2 (unpaired t-test, wt vs. tg, p = 0.57, wt vs. tg/tg, p = 0.015) but were comparable to controls at day 3. In contrast, naïve ARTE10 mice were indistinguishable from controls at 12 months of age. In the probe trial memory for the platform location was determined. At 4 and 8 months of age all animals displayed good memory as they swam significantly over chance level (12.5%) in their respective target zones (Fig. 6b, p<0.05 for all measures). By 12 months, mice of the longitudinal cohort were indistinguishable from controls in memory retrieval. In contrast, mice from the cross-sectional cohort displayed poor memory as their preference for the target zone was borderline non-significant over chance level (one sample t-test, hypothesized mean ≤12.5%; wt, p = 0.01; tg, p = 0.052; tg/tg, p = 0.052).

Episodic memory of mice from the cross-sectional cohorts was examined in the object recognition test. Eight months old mice displayed good memory of a previously explored object, indicated by higher preference for a new versus the old object (Fig. 6c). By the age of 12 months a deficit in episodic memory was evident in homozygous ARTE10 mice as exploration of the new object was not significant from chance level (one sample t-test, hypothesized mean ≤50%; wt, p = 0.0004; tg, p = 0.002; tg/tg, p = 0.293).

### Treatment with a γ-Secretase Inhibitor

**Figure 7:**
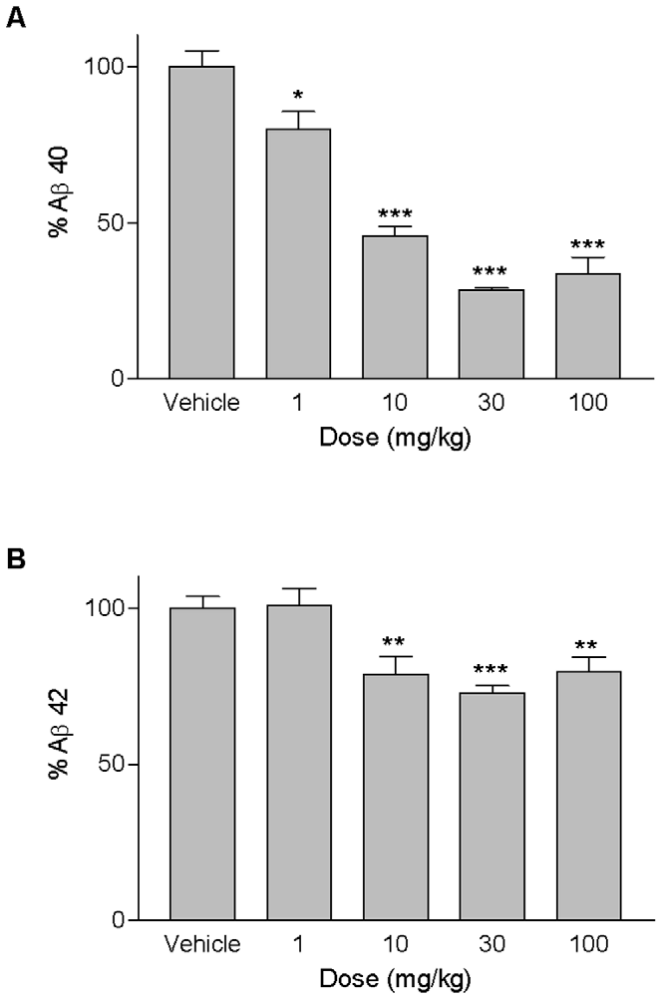
Effect of a γ-secretase inhibitor on soluble brain Aβ40 and Aβ42. MRK-560/compound 32 was applied p.o. to hemizygous transgenic mice and brain Aβ levels were analysed 4 h post-dosing. Soluble Aβ40 (A) and Aβ42 (B) were significantly reduced after treatment with the γ-secretase inhibitor. Data are expressed as means + SEM. (*) p = 0.0176, (**) p<0.005, (***) p<0.0001 versus vehicle.

The γ-secretase inhibitor MRK-560/compound 32 [11] was used in order to evaluate the ARTE10 model for its use in assessing β-amyloid lowering therapies. After acute dosing with the inhibitor levels of both soluble Aβ40 and Aβ42 were significantly reduced in the brains of ARTE10 mice (Fig. 7). Aβ40 levels were reduced dosage-dependent up to 72% with an ED50 of 2.7 mg/kg, whereas Aβ42 secretion could only be inhibited by about 27%.

## Discussion

The ARTE10 mouse model exhibits a neuropathological phenotype that mimics several characteristics of human AD. In particular, ARTE10 mice develop AD-like cerebral β−amyloidosis with respect to the morphology and composition of amyloid plaques as well as their progressive spatial and temporal pattern of distribution. Emulating the classical tripartite morphology of senile plaques in human AD [14], the plaques of ARTE10 mice invariably consist of the three principal components: (1) a dense core deposit of congophilic Aβ in the centre, surrounded by (2) numerous ubiquitin-positive dystrophic neurites and by (3) glial inflammation represented by reactive astrocytes and activated microglia. Individual plaques sizes and maximum cortical plaque burden are similar to the respective findings in human AD [6], [15]. The absence of full-blown neurofibrillary changes, i.e. argyrophilic neurites, neurofibrillary tangles or neuropil threads, is in line with other transgenic mouse models based on the (co-)expression of APP and presenilin mutants which also lack neurofibrillary pathology. However, homozygous ARTE10 animals aged 8 months or older show some nascent tau hyperphosphorylation in few plaque-associated dystrophic neurites. Similar findings have only been reported in a few other APP-PS1 mouse models [16], [17]. The accumulation of ubiquitin is involved in the development of neurofibrillary changes [18].

We found intracellular Aβ in several pyramidal neurons of the cortex and hippocampus already in young ARTE10 mice that had not yet developed plaques. The speckled distribution of Aβ immunoreactivity within the neuronal perikarya closely resembles the intracellular Aβ immunoreactive pattern documented for human AD as well as some other murine AD models, where it is detectable in vulnerable brain regions preceding plaque deposition and the development of neurofibrillary changes [19], [20]. Interestingly, we could show a similar pattern of intracytoplasmic immunoreactivity by the anti-oligomer conformation-specific antibody A11 in some neurons, suggesting that intraneuronal Aβ is at least in part present in an oligomeric state early-on in ARTE10 mice (Fig. S6).

Plaque deposition starts at 3 and 5 months respectively in homozygous and hemizygous ARTE10 mice. Plaque burden is steadily progressive and follows a saturation curve. The spatial and temporal distribution of plaques primarily and predominantly involves the hippocampus, the neocortex, and limbic cortical areas such as the amygdala. Subcortical structures are affected later and the cerebellum seems to be spared even in aged mice. This pattern and sequence of brain regions is equivalent to the spatio-temporal spread of AD pathology in the human brain [21]–[24]. Interestingly, the plaque morphology, distribution pattern and sequence of plaque formation in ARTE10 mice are relatively similar to some of the other APP and presenilin expressing mouse lines, although in some cases different promoters and even different APP and/or presenilin mutants have been used [2], [4], [25], [26]. However, in some models like APPPS1-21 and Tg2576 mice, amyloid plaques could also be observed in the cerebellum ([16], unpublished observation), which is neither the case in ARTE10 mice nor human patients. Amyloid angiopathy of intracortical, leptomeningeal and choroidal blood vessels is a regular concomitant of human AD [14], and it is found likewise in ARTE10 animals with advancing amyloidosis. This is in line with established transgenic mouse models in which both parenchymal and vascular amyloidosis could be found [27]. Other models were described that exhibit either predominantly parenchymal [16] or only vascular amyloid [28].

Aβ42 constitutes the predominant insoluble Aβ species in homozygous as well as hemizygous ARTE10 mice, which is in accordance with other transgenic lines co-expressing APPswe and mutant PS1 [29], [30]. This probably accounts for the early and robust amyloid deposition in ARTE10 mice. Accordingly, higher ratios of Aβ42 over Aβ40 were also observed in human patients with PS1 mutations [31].

We observed one difference between ARTE10 mice and human AD: Whereas in human brain diffuse plaques precede mature amyloid plaques [32], in ARTE10 brain, inversely, diffuse Aβ deposits only became detectable as small “satellites” in proximity to substantial amounts of mature plaques. Taking advantage of this, the model is particularly qualified for the purpose of in vivo validation of substances mainly interacting with mature amyloid plaques, e.g. radiotracers for in vivo diagnostic imaging and quantification of amyloid plaques in patients' brains [12], [33]. Here, we have demonstrated visualization of plaques in ARTE10 brain ex vivo after intravenous injection of a radiotracer. An in vivo PET study of Aβ in ARTE10 mice using [^11^C]-labeled PIB is reported separately (Manook et al, submitted).

The onset, development and maximal amount of amyloid plaque deposition vary considerably between established mouse lines (co-) expressing APP and presenilin mutants [3]. The homozygous ARTE10 is among those lines displaying a relatively early plaque onset and a high maximal plaque burden (table 1)[16], [26], [29], [34]–[37]. However, direct comparisons of published data are very difficult as quantification methods have not been standardized and studies comparing different mouse lines are largely missing. 100% penetrance of the plaque phenotype is rarely reported in the literature but is an important feature of a model for the use in amyloid-lowering experiments and likewise a measure of variability. By 5 months of age, two months after plaque onset, the brains of all homozygous ARTE10 mice examined exhibited amyloid plaques. Only for TgCRND8 mice expressing the APP with swedish and indiana mutations 100% penetrance was reported at an earlier age [26]. The variability in plaque load between mice of the same age is relatively low in homozygous ARTE10 mice but comparable to other models expressing both APP and PS1 at an equivalent age. For several AD models gender differences have been reported but were not observed in the hemi-as well as homozygous ARTE10 line [34], [38]–[41].

**Table 1:** Comparison of amyloid burden in transgenic mouse models of AD.

Mouse model	Transgenes^a^	Plaque onset	100% penetrance	Plaque load in aged miceb, c	Ref.
Tg2576	PrP-APP_swe_	∼9 m (6–11 m)	11–13 m	6.077+/− 0.99 (24 m)	[2], [35], [65]
APP23	thy1-APP_swe_	6–9 m	18 m	24.1+/− 0.9% (15.9–28%, 27 m)	[27], [66], [67]
TgCRND8	PrP-APP_swe,ind_	2–3 m	3 m	∼4% (9–11 m)	[26], [63]
Tg2576 x	PrP-APP_swe_ x	3 m	n.r.	Cx: 52% (28–64%, 15 m)	[68], [69]
PS1 5.1	PDGF-PS1_M146L_			Hx: 25% (10–42%, 15 m)	
APPPS1-21	thy1-APP_swe_ +	Cx: 1.5 m	n.r.	Cx: 20.9+/− 0.8% (19 m)	[16]
	thy1-PS1_L166P_ d	Hx: 2–3 m		Hx: 9.6+/− 0.4% (19 m)	
PS2APP	thy1-APP_swe_ x PrP-PS2_N141I_	5 m	n.r.	5.5+/− 1.5% (17–18 m)	[4]
ARTE10	thy1-APP_swe_ +	5 m	10 m	10.5+/− 2.2%	This
(tg)	thy1-PS1_M146V_ d			(0.2–35.6%, 19–20 m)	paper
ARTE10	thy1-APP_swe_ +	3 m	5 m	35.2+/− 2.8%	This
(tg/tg)	thy1-PS1_M146V_ d			(26.4–53.2%, 19–20 m)	paper

cx, cortex; hx, hippocampus; m, months; n.r., not reported; PrP, prion protein; tg, hemizygous; tg/tg, homozygous.

a promoter-cDNA.

b +/− SEM, range and age in brackets.

c in cortex and hippocampus.

d co-injected.

ARTE10 mice can be crossed and maintained as double homozygous line for both APP and PS1 transgenes which has, to the best of our knowledge, not been described before. Intercrossing of the line to homozygosity was facilitated by the heredity co-transmission of the APP and PS1 transgenes. Also, crossing to gene-targeted mice, for the validation of drug targets directly on an AD background [42], or to other transgenic lines, e.g. tau expressing mice, thereby creating a mouse model comprising of both plaques and tangles, may be simplified. The breeding performance and viability of ARTE10 mice, backcrossed to C57BL/6, is undistinguishable from wild type mice, even as homozygous line. We observed 15% mortality in homozygous mice at 12 months which is very low in comparison to reports from other established AD models, as far as data are published. Premature death of up to 50% has been reported for the widely used Tg2576 and TgCRND8 mice on C57Bl/6 background at 12 and 9 months respectively [26], [43]. Others describe mortality between 21–70% dependent on expression levels in different APP_swe_ lines at 12 months of age [44].

We have developed and used a novel image analysis algorithm for the quantitative assessment of the plaque load, number per area and size distribution of amyloid plaques in defined regions of the mouse brain. These morphological parameters can be of importance in amyloid-lowering therapies, e.g. in which the plaque number appears to be more sensitive than total amyloid burden [45]. Different attempts have been made to implement computer-assisted analysis for quantification of amyloid plaques on human and mouse brain sections [25], [46] . A frequent difficulty of image analysis is identification of false positive structures arising from background staining and tissue artefacts, thereby lowering the specificity and reliability of object recognition. The here described algorithm based on the Acapella™ image analysis software implements multi-channel segmentation and dynamic thresholding for elimination of tissue background and artefacts thus outperforming the common global threshold based methods [25] . In order to test the validity and reliability of our novel Acapella procedure, morphological and biochemical analyses were conducted in parallel on ARTE10 mice at different ages. Plaque loads were determined by Acapella in one brain hemisphere, and levels of insoluble Aβ extracted with formic acid from the contralateral brain hemisphere of the same animal were measured by ELISA. The quantitative results of the two different approaches strongly correlated with each other. Thus, the validity and reliability of our novel morphological procedure have been confirmed successfully by the use of an independent, conventional ELISA method.

Synaptic loss and dysfunction already occur at very early stages of AD [47], [48] mirrored by a decrease of pre- and post-synaptic marker proteins synaptophysin [49]–[51], Disk large homolog 4 (DLGH4 [52]) and Drebrin (DBN1 [53]–[55]). Whereas a direct spatial relationship of amyloid plaque deposition to synaptic loss was shown in AD mouse models [56]–[58], synaptic deficits have been shown to also occur prior to plaque deposition [59], [60]. Analyses of ARTE10 mouse brains at different points of age revealed decreased mRNA levels of all three synaptic marker proteins already at 3–4 months of age. Such decreased mRNA levels are of the same magnitude in both hemi- and homozygous ARTE10 mice in contrast to the respective gene dose-dependent differences in the amount and onset of plaque deposition. This observation is in accordance with early-onset synaptic defects as described in other AD mice [59], [60].

By the age of 12 months, ARTE10 mice developed cognitive deficits mainly in episodic memory, which is in accordance with observations made in other mouse models [61]. A subtle spatial learning deficit was only observed in mice of the longitudinal group that were tested repeatedly and can be explained by an impaired flexibility of transgenic mice as has been found in other transgenic APP-PS1 mice [16] and human AD patients [62]. At the age when cognitive deficits occur, the plaque load has already reached an advanced stage implying a direct link between plaque deposition and cognitive impairment as suggested in previous studies [63]. Other studies showed a correlation between cognitive deficits and soluble, oligomeric forms of Aβ as explanation for cognitive deficits before the appearance of first plaques [64]. However, analyses with human AD patients are largely missing and needed to clarify the role of Aβ in this process.

Finally, levels of soluble Aβ in the brain could be reduced by acute treatment of ARTE10 mice with a γ-secretase inhibitor. In accordance with the literature we could find a dosage-dependent inhibition of Aβ40 secretion by maximal 75% [11]. Aβ42 levels were reduced by 25% which has not been reported before for this γ-secretase inhibitor. In summary, these results demonstrate the suitability of the transgenic model in the development and exploration of amyloid-lowering therapies.

In conclusion, the double-transgenic APP-PS1 mouse line ARTE10 unifies several features for a meaningful and easy to use model for AD-like cerebral β-amyloidosis i) an early-onset and rapid progression of plaque load, ii) plaques showing similar morphology to those in human AD, iii) low inter-animal variability and no gender effects, iv) the co-inherited transgenes and a C57BL/6 background facilitate breeding to other transgenic or gene-targeted mice, v) good breeding capabilities of the homozygous line and a low rate of premature death, vi) early loss of synaptic markers and age-related cognitive deficits. Together with the described plaque load analysis and as demonstrated by using a γ-secretase inhibitor, ARTE10 is well suited for testing of Aβ-modulating treatment protocols and may also be of use in target validation approaches.

## Supporting Information

Supporting File S1 Supplemental results and methods.
(0.02 MB PDF)

Figure S1 Specific immunofluorescent detection of the transgenic human, but not endogenous murine, APP protein in ARTE10 mouse brains with the human-specific anti-APP N-terminal mouse monoclonal antibody LN27. The transgenic human protein is clearly detected in a neuronal pattern on brain sections from an 8 months old homozygous female (A–C), a 4 months old hemizygous female (G–K), and a 4 months old hemizygous male (L–N) mouse. Endogenous mouse APP is not labelled on brain sections from an 8 months old female wild type littermate (D–F). (A, D, G, L) frontal neocortex. (B, E, H, M) posterior neocortex, parts of the subiculum and the CA1 region of the hippocampus. (C, F, K, N) hippocampus. All micrographs were taken and are shown at the same magnification. Scale bar in (A), 200 µm.
(4.99 MB TIF)

Figure S2 Plaque detection with Acapella™. (A) Digital micrograph of a mouse brain section stained against amyloid β. Regions of interest (neocortex and hippocampus) are marked manually. (B) Original image reduced to neocortex and hippocampus. (C) Image is split into hue, saturation, and value (HSV) channels. (D) The image analysis algorithm detects stained structures by individually segmenting the HSV channels. Channel results are connected in order to retrieve plaque load, size, and number. (E) Outcome of plaque detection visualized with green labels. For details please refer to the Methods section.
(5.13 MB TIF)

Figure S3 Double-immunofluorescent detection of Aβ40 (green) and Aβ42 (red) in the cortex of a 10 months-old homozygous ARTE10 mouse (blue, nuclei/DAPI). Most plaques are dense-cored and contain both Aβ40 and Aβ42 species, resulting in merged yellow color. The arrows point to some diffuse deposits consisting of Aβ42 (for details, see text). Scale bars: A, 200 µm; B, 50 µm.
(8.70 MB TIF)

Figure S4 Left column: Thioflavin-S positive plaque cores (green), each encircled by a sphere (corona) of dilated, strongly ubiquitin-positive dystrophic neurites (red) in the cortex of four homozygous ARTE10 mice between 5 and 13 months of age. Right column: Triple-immunofluorescent demonstration of plaque-associated mixed glial inflammation: Activated microglia (green, AIF1/Iba1) and reactive astroglia (magenta, GFAP) colonizing the periphery of amyloid cores (red: Aβ/6E10) in the cortex of four homozygous ARTE10 mice between 5 and 13 months of age (blue: nuclei/DAPI). Scale bars, 50 µm each.
(9.86 MB TIF)

Figure S5 Triple-immunofluorescent detection of the pre-synaptic marker protein synaptophysin (green) accumulating in plaque-associated swollen dystrophic neurites of the plaque corona, apart from its physiological localization in the neuropil. Co-staining with the somato-dendritic marker MAP2 (magenta), with human APP (red) and nuclear staining with DAPI (blue). A, Subiculum of a 9 months-old hemizygous ARTE10 mouse. B, Dentate gyrus of a 7 months-old homozygous ARTE10 mouse. C, frontal cortex of an 8 months-old homozygous ARTE10 mouse. Scale bar for all three images, 50 µm.
(10.00 MB TIF)

Figure S6 Immunofluorescent detection of intracellular Aβ (green) by 4G8 (A, B) or AB1 (C) in cortical neurons, shown here in two young ARTE10 animals (1 and 3 months). APP is co-stained in red in the left two images. The speckled intracytoplasmic Aβ immunostaining is paralleled by virtually the same pattern detected by the anti-oligomer conformation-specific antibody A11 (green, D) within the perikarya. This pattern closely resembles the intracellular Aβ immunoreactivity of affected neurons in human AD as well as in other murine AD models and suggests that intraneuronal Aβ is at least in part present in an oligomeric state early-on in ARTE10 mice. Scale bar in (A), 10 µm.
(4.51 MB TIF)

Figure S7 Thioflavin-S staining (green, FITC channel) for fluorescent detection of amyloid plaques in parasagittal brain sections of representative hemi- (tg) and homozygous (tg/tg) ARTE10 mice at different ages (3, 8, 12, and 20 months), illustrating the effects of age and gene dose on the plaque load. Tissue autofluorescence recorded in the red/Cy3 channel (in the absence of fluorophore) is utilized for the morphological visualization of gross anatomical structures. The sections are always oriented with the cortex at the top, the anterior part at the left, the posterior part at the right. Fluorescent micrographs were recorded separately for each channel by means of a Olympus BX51 microscope using the appropriate dichroic mirrors and filters, a 2x objective, a 0.5x TV adapter, and a ColorView-II CCD camera. The channels were merged using the Olympus analySIS “FIVE” imaging software. Parallel sections from the same animals as in Figure S8 are shown.
(9.56 MB TIF)

Figure S8 Immunohistochemical detection of Aβ (6E10, green) in parasagittal brain sections of representative hemi- (tg) and homozygous (tg/tg) ARTE10 mice at different ages (3, 8, 12, and 20 months), illustrating the effects of age and gene dose on the plaque load.
(10.17 MB TIF)

Figure S9 Statistical analysis of synaptic marker mRNA expression analysis. A, Synaptophysin (Syp), B, Disk large homolog 4 (Dlgh4), and C, Drebrin (Dbn1) mRNA expression in hemizygous and homozygous ART10 mice were analyzed in comparison to wild type animals. Material from comparable brain regions of mice ranging from 3 to 13 months of age was used for each of these studies. mRNA has been extracted from the brain material and expression of Synaptophysin, Disk large homolog 4, and Drebrin was measured applying real-time quantitative PCR. For normalization, each ratio of Synaptophysin, Disk large homolog 4, and Drebrin values with Cyclophilin B (Ppib) have been calculated. In comparison to wild type mice hemi- as well as homozygous mice revealed a significantly lower mRNA level of Synaptophysin (p = 0.0011; 0.0003), Disk large homolog 4 (p = 0.0003; 0.0011), and Drebrin (p = 0.0003; 0.0002), respectively; statistical analyses were done by Mann-Whitney U-test.
(0.10 MB TIF)

Table S1 Results of tests examining body weight, body temperature and motor-sensory abilities of hemizygous and homozygous transgenic mice in comparison to wild type littermates. Data are expressed as means +/− SEM; * p<0.05 versus wild type littermates (Wt); Tg hemizygous transgenic mice; Tg/tg homozygous transgenic mice.
(0.01 MB PDF)

Table S2 Results of behavioural tests examining locomotor and exploratory activity, anxiety and spontaneous alternation of heterozygous and homozygous transgenic mice in comparison to wild type littermates. Data are expressed as means +/− SEM; * p<0.05 versus wild type littermates (Wt); # p<0.05 versus hemizygous littermates (Tg); Tg/tg homozygous transgenic mice.
(0.01 MB PDF)
