# Supplementary material for: Early-Onset and Robust Amyloid Pathology in a New Homozygous Mouse Model of Alzheimer's Disease
Source: PLoS One. 2009 Nov 20;4(11):e7931. doi: 10.1371/journal.pone.0007931 (PMC2775952; doi:10.1371/journal.pone.0007931)
Supplement: Supporting File S1 — Supplemental results and methods. (0.02 MB PDF) [file pone.0007931.s001.pdf]

SUPPORTING INFORMATION

Supplemental Results

Sensorimotor, locomotory and exploratory behavior

In tests examining the animals´ health and simple reflexes, mice from all groups appeared healthy at all time points measured without differences between groups in body weight, body temperature and other health parameters (Table S1 and data not shown). The sensorimotor abilities of ARTE10 mice tested in grip strength, rotarod and hot plate test were similar to controls, except that homozygous transgenic mice showed a tendency towards decreased heat sensitivity which appeared statistically significant only in 8 months old mice from the longitudinal cohort (one-way ANOVA, $F_{(2,41)} = 3.51$, p = 0.039; post-hoc analysis wt vs. tg/tg, p = 0.012). During rotarod testing in all cohorts and at all time points, an effect of gender but not of genotype on motor-coordination could be observed (Table S1).

Open field and zero maze tests were performed in order to measure locomotor and exploratory behavior of the mice as well as anxiety (Table S2). At 4 months of age homozygous ARTE10 mice spent less time in the centre of the open field (one-way ANOVA, $F_{(2,40)} = 5.03$, p = 0.011; post-hoc analysis wt vs. tg/tg, p = 0.003, tg vs. tg/tg, p = 0.040) and entered the open sector of the zero maze less often than their wild type litter mates (one-way ANOVA, $F_{(2,40)} = 3.59$, p = 0.037; post-hoc analysis wt vs. tg/tg, p = 0.012), suggestive of an increased anxiety phenotype. However, at older ages and in the cross-sectional cohorts no differences could be observed between transgenic and wild type animals in the open field and zero maze. The locomotor activity of ARTE10 mice during 30 min in the open field appeared similar to wild type controls throughout the whole testing period. However, in Y maze the number of arm entries was consistently reduced in ARTE10 mice at 4 months and in 8 and 12 months old animals in the cross-sectional study which was less obvious in the experienced mice of the longitudinal study (Table S2). Spontaneous alternation, a measure of spatial working memory, remained unchanged in all cohorts except at 8 months in the longitudinal study where a borderline significance was reached (one-way ANOVA, $F_{(2,41)} = 3.25$, p = 0.049; post-hoc analysis wt vs. tg/tg, p = 0.027, tg vs. tg/tg, p = 0.036).

In summary, young but not aged transgenic mice displayed an increased anxiety, paralleled by a decrease in exploratory activity, which is in accordance with previous reports showing

neophobia and decreased exploration in other mouse models of AD [1–3]. In addition, ARTE10 mice show a tendency towards decreased locomotor activity, which was mainly evident by a reduced number of arm visits in the Y maze. Decreased locomotion was also observed by [4] whereas others reported hyperactivity in transgenic AD mouse models [2,5,6].

Supplemental Methods

Behavioral analyses.

Tests for neurological and sensorimotor function: A series of tests were performed in order to check the animals´ overall health, simple reflexes and sensorimotor function. These included examination of body weight, body temperature, coat appearance, secretory signs, body posture, eye blink reflex, pupil constriction and dilation, flexion reflex, righting reflex, grip strength test (adapted from the SHIRPA test battery [7–9]). The accelerating rotarod (Ugo Basile) test was performed as described [10]. Briefly, mice were trained to stay on the rod in two training sessions for 3 min each at low constant speed (4 rpm). 3 trials were carried out with accelerating rod (4 – 40rpm) for maximal 5 min. A forth trial was performed the following day with accelerating rod for 5 min. For grip strength test mice were forced to pull on a strain gauge and releasing points are recorded. The mean of 3 trials was calculated. The hot plate test was performed according to [11]. The temperature of the hot plate was set at 52°C and the latency until the animals showed any signs of heat sensation was recorded.

Tests for locomotor activity, exploration and anxiety: Locomotor and exploratory activity was measured by placing the animals for 30 min into an open field box (50 x 50 x 50 cm). Locomotor and exploratory activity was recorded and analyzed using a video camera and EthoVision© Software (Noldus). The area of the open field was divided in an outer zone (home zone) and an inner zone (center) of 40 x 40 cm. Since the central region of an open field is often regarded to be more anxiogenic than the outer rim exploratory activity was measured in the center of the open field within the first 10 min of the test.

Anxiety was measured using an elevated zero maze as described [12].The maze was made of grey plastic and had an annular runway (diameter 46 cm, width 5.5 cm). Two opposing 90° sectors of the runway were protected by an inner and outer wall of grey polyvinyl-chloride (height 16 cm). The two remaining sectors were unprotected. Animals were placed in one of the protected zones of the maze. % time spent on open sectors and the % of entries in open sectors were recorded for 5 min by a video camera and EthoVision© Software (Noldus). Numbers of head dips (protected and unprotected) were recorded manually. Data analysis was done using Wintrack Software [13].

Y maze: The spontaneous alternation task was performed in a Y-maze made of grey plexiglass. Each arm was 40 cm long, 20 cm high, 10 cm wide. The arms converged in an equilateral triangular central area. Each mouse was placed at the end of one arm and allowed

to move freely through the maze during a 5-min session. The series of arm entries was recorded manually. Arm entry was considered to be completed when the hind paws of the mouse had been completely placed in the arm. Alternation was defined as successive entries into the three arms, in overlapping triplet sets. The percent alternation was calculated as the ratio of actual to possible alternations (defined as the total number of arm entries – 2) multiplied by 100%.

Supplemental References

1. Moechars D, Dewachter I, Lorent K, Reverse? D, Baekelandt V, Naidu A, Tesseur I, Spittaels K, Van Den Haute C, Checler F, Godaux E, Cordell B, Van Leuven F (1999) Early phenotypic changes in transgenic mice that overexpress different mutants of amyloid precursor protein in brain. Journal of Biological Chemistry 274: 6483-6492.

2. Dodart JC, Meziane H, Mathis C, Ungerer A, Bales KR, Paul SM (1999) Behavioral disturbances in transgenic mice overexpressing the V717F beta-amyloid precursor protein. Behavioral Neuroscience 113: 982-990.

3. Lee KW, Lee SH, Kim H, Song JS, Yang SD, Paik SG, Han PL (2004) Progressive Cognitive Impairment and Anxiety Induction in the Absence of Plaque Deposition in C57BL/6 Inbred Mice Expressing Transgenic Amyloid Precursor Protein. Journal of Neuroscience Research 76: 572-580.

4. Van Dam D, D'Hooge R, Staufenbiel M, Van Ginneken C, Van Meir F, De Deyn PP (2003) Age-dependent cognitive decline in the APP23 model precedes amyloid deposition. European Journal of Neuroscience 17: 388-396.

5. Richards JG, Higgins GA, Ouagazzal AM, Ozmen L, Kew JNC, Bohrmann B, Malherbe P, Brockhaus M, Loetscher H, Czech C, Huber G, Bluethmann H, Jacobsen H, Kemp JA (2003) PS2APP transgenic mice, coexpressing hPS2mut and hAPPswe, show age-related cognitive deficits associated with discrete brain amyloid deposition and inflammation. Journal of Neuroscience 23: 8989-9003.

6. King DL, Arendash GW (2002) Behavioral characterization of the Tg2576 transgenic model of Alzheimer's disease through 19 months. Physiology and Behavior 75: 627-642.

7. Rogers DC, Fisher EMC, Brown SDM, Peters J, Hunter AJ, Martin JE (1997) Behavioral and functional analysis of mouse phenotype: SHIRPA, a proposed protocol for comprehensive phenotype assessment. Mammalian Genome 8: 711-713.

8. Higgins GA, Grottick AJ, Ballard TM, Richards JG, Messer J, Takeshima H, Pauly-Evers M, Jenck F, Adam G, Wichmann J (2001) Influence of the selective ORL1 receptor agonist, Ro64-6198, on rodent neurological function. Neuropharmacology 41: 97-107.

9. Crawley JN, Paylor R (1997) A proposed test battery and constellations of specific behavioral paradigms to investigate the behavioral phenotypes of transgenic and knockout mice. Hormones and Behavior 31: 197-211.

10. Freitag S, Schachner M, Morellini F (2003) Behavioral alterations in mice deficient for the extracellular matrix glycoprotein tenascin-R. Behavioural Brain Research 145: 189-207.

11. Mobarakeh JI, Sakurada S, Katsuyama S, Kutsuwa M, Kuramasu A, Lin ZY, Watanabe T, Hashimoto Y, Watanabe T, Yanai K (2000) Role of histamine H1 receptor in pain perception: A study of the receptor gene knockout mice. European Journal of Pharmacology 391: 81-89.

12. Konig M, Zimmer AM, Steiner H, Holmes PV, Crawley JN, Brownstein MJ, Zimmer A (1996) Pain responses, anxiety and aggression in mice deficient in pre- proenkephalin. Nature 383: 535-538.

13. Wolfer DP, Madani R, Valenti P, Lipp HP (2001) Extended analysis of path data from mutant mice using the public domain software Wintrack. Physiology and Behavior 73: 745-753.
