# Supplementary figures and images for: Early-Onset and Robust Amyloid Pathology in a New Homozygous Mouse Model of Alzheimer's Disease
Source: PLoS One. 2009 Nov 20;4(11):e7931. doi: 10.1371/journal.pone.0007931 (PMC2775952; doi:10.1371/journal.pone.0007931)

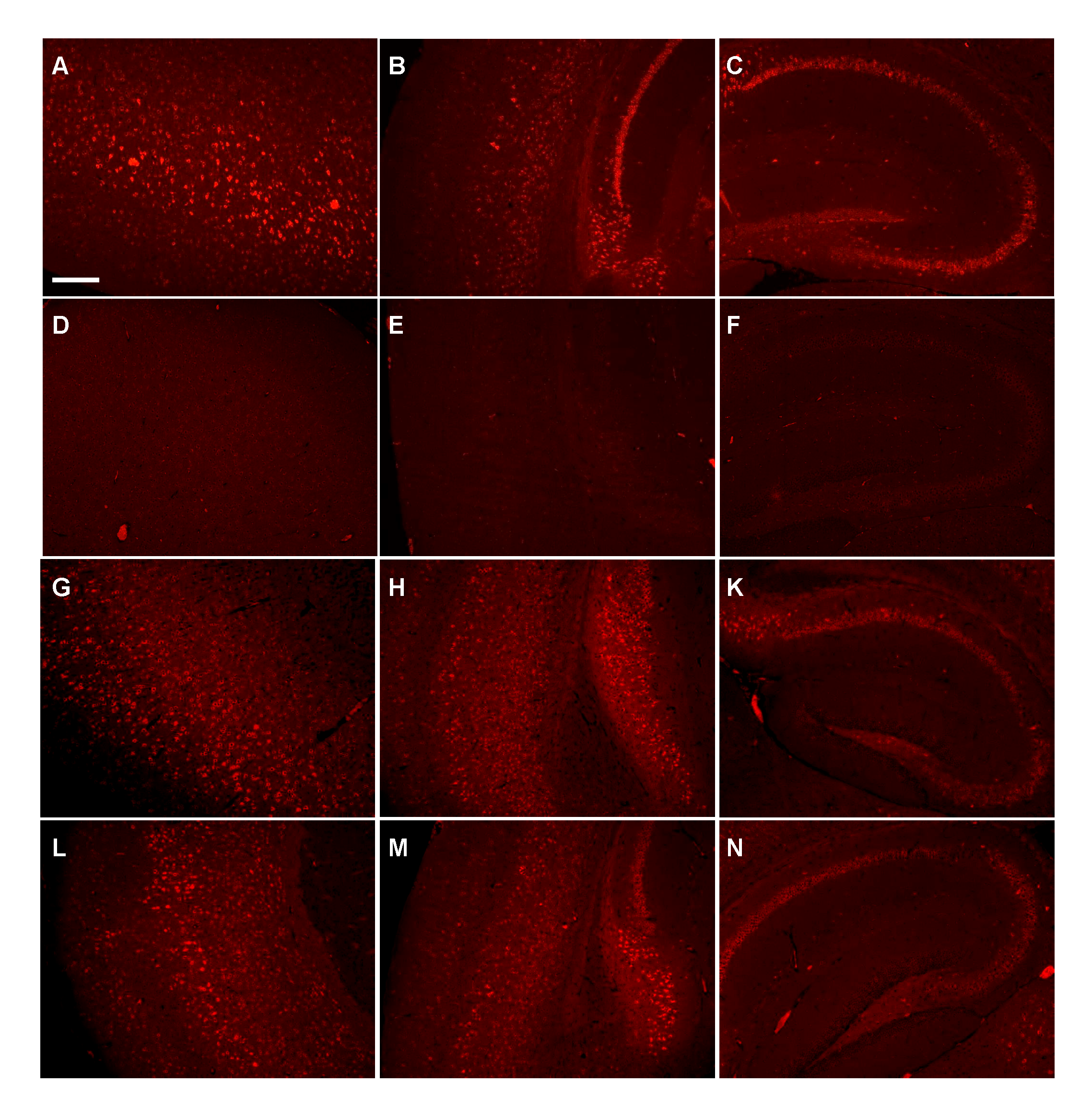

Supplement: Figure S1 — Specific immunofluorescent detection of the transgenic human, but not endogenous murine, APP protein in ARTE10 mouse brains with the human-specific anti-APP N-terminal mouse monoclonal antibody LN27. The transgenic human protein is clearly detected in a neuronal pattern on brain sections from an 8 months old homozygous female (A–C), a 4 months old hemizygous female (G–K), and a 4 months old hemizygous male (L–N) mouse. Endogenous mouse APP is not labelled on brain sections from an 8 months old female wild type littermate (D–F). (A, D, G, L) frontal neocortex. (B, E, H, M) posterior neocortex, parts of the subiculum and the CA1 region of the hippocampus. (C, F, K, N) hippocampus. All micrographs were taken and are shown at the same magnification. Scale bar in (A), 200 µm. (4.99 MB TIF) [file pone.0007931.s002.tif]

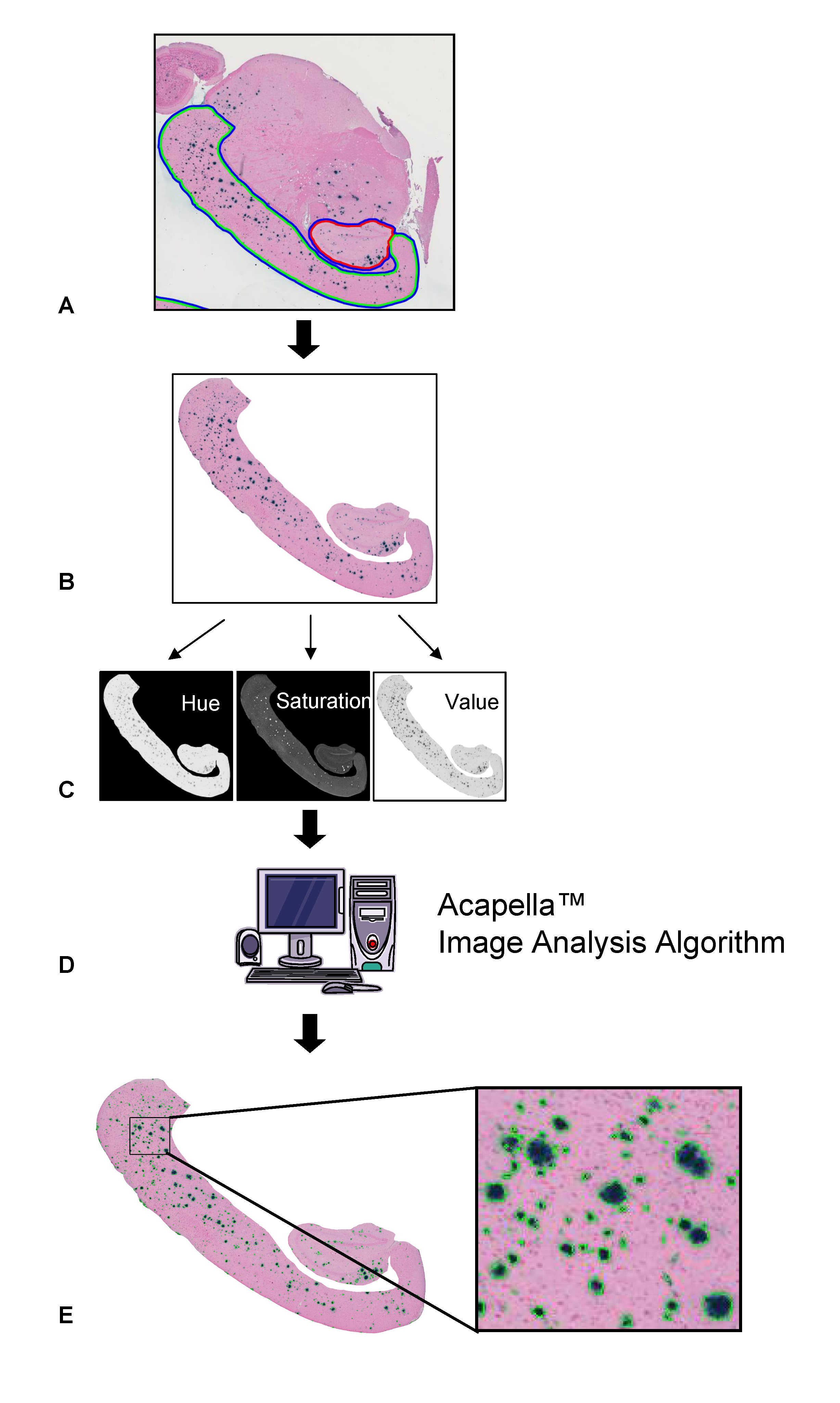

Supplement: Figure S2 — Plaque detection with Acapella™. (A) Digital micrograph of a mouse brain section stained against amyloid β. Regions of interest (neocortex and hippocampus) are marked manually. (B) Original image reduced to neocortex and hippocampus. (C) Image is split into hue, saturation, and value (HSV) channels. (D) The image analysis algorithm detects stained structures by individually segmenting the HSV channels. Channel results are connected in order to retrieve plaque load, size, and number. (E) Outcome of plaque detection visualized with green labels. For details please refer to the Methods section. (5.13 MB TIF) [file pone.0007931.s003.tif]

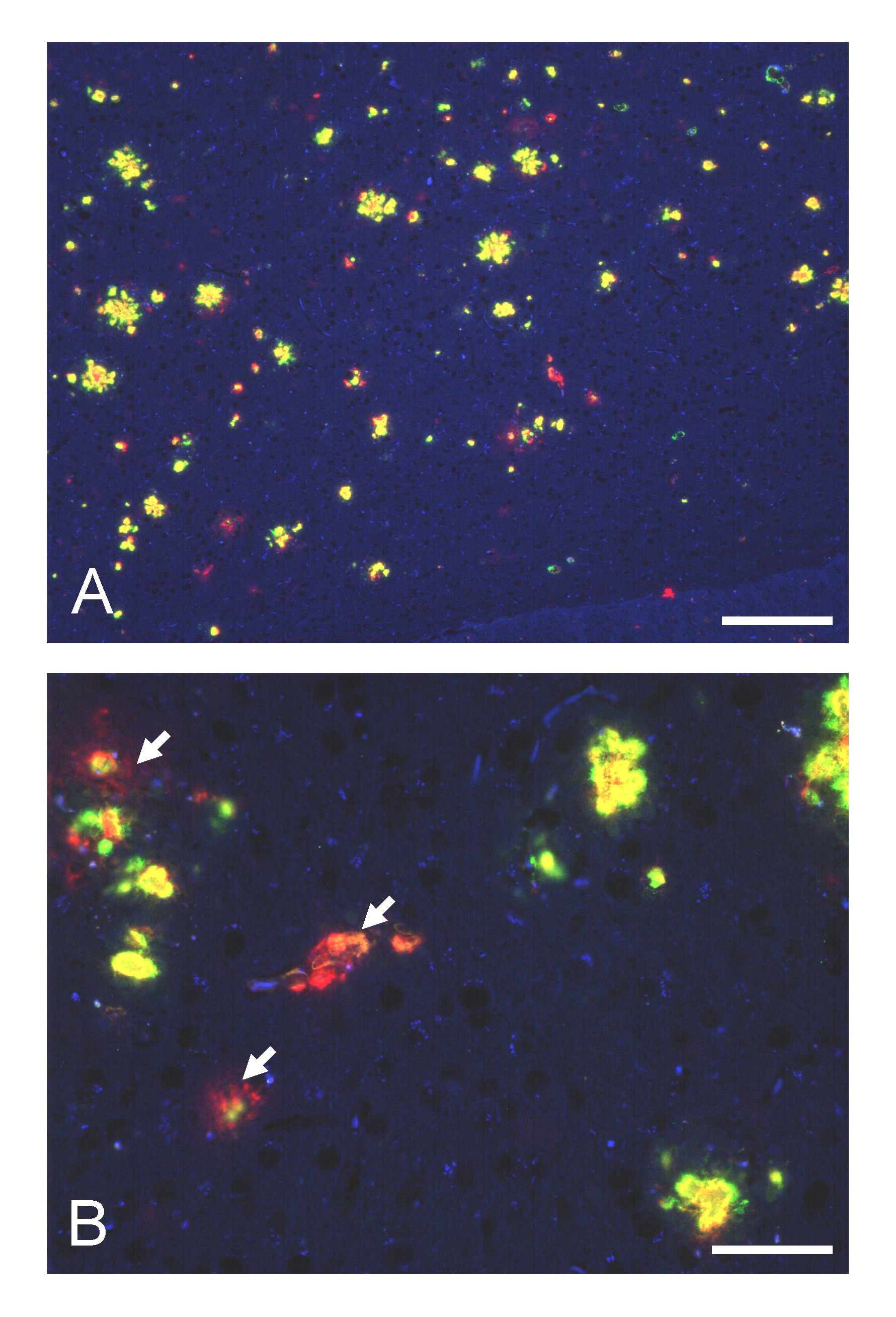

Supplement: Figure S3 — Double-immunofluorescent detection of Aβ40 (green) and Aβ42 (red) in the cortex of a 10 months-old homozygous ARTE10 mouse (blue, nuclei/DAPI). Most plaques are dense-cored and contain both Aβ40 and Aβ42 species, resulting in merged yellow color. The arrows point to some diffuse deposits consisting of Aβ42 (for details, see text). Scale bars: A, 200 µm; B, 50 µm. (8.70 MB TIF) [file pone.0007931.s004.tif]

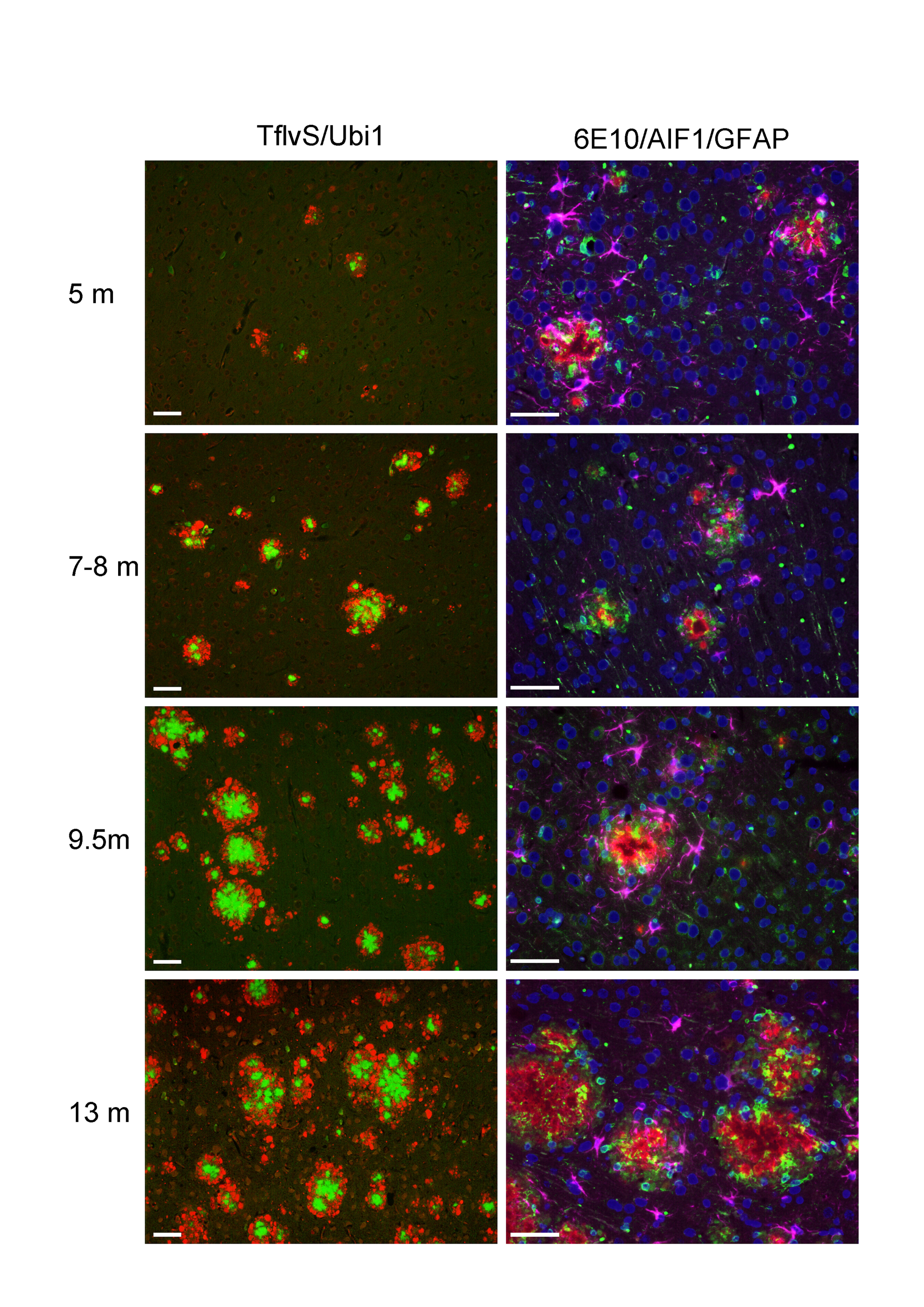

Supplement: Figure S4 — Left column: Thioflavin-S positive plaque cores (green), each encircled by a sphere (corona) of dilated, strongly ubiquitin-positive dystrophic neurites (red) in the cortex of four homozygous ARTE10 mice between 5 and 13 months of age. Right column: Triple-immunofluorescent demonstration of plaque-associated mixed glial inflammation: Activated microglia (green, AIF1/Iba1) and reactive astroglia (magenta, GFAP) colonizing the periphery of amyloid cores (red: Aβ/6E10) in the cortex of four homozygous ARTE10 mice between 5 and 13 months of age (blue: nuclei/DAPI). Scale bars, 50 µm each. (9.86 MB TIF) [file pone.0007931.s005.tif]

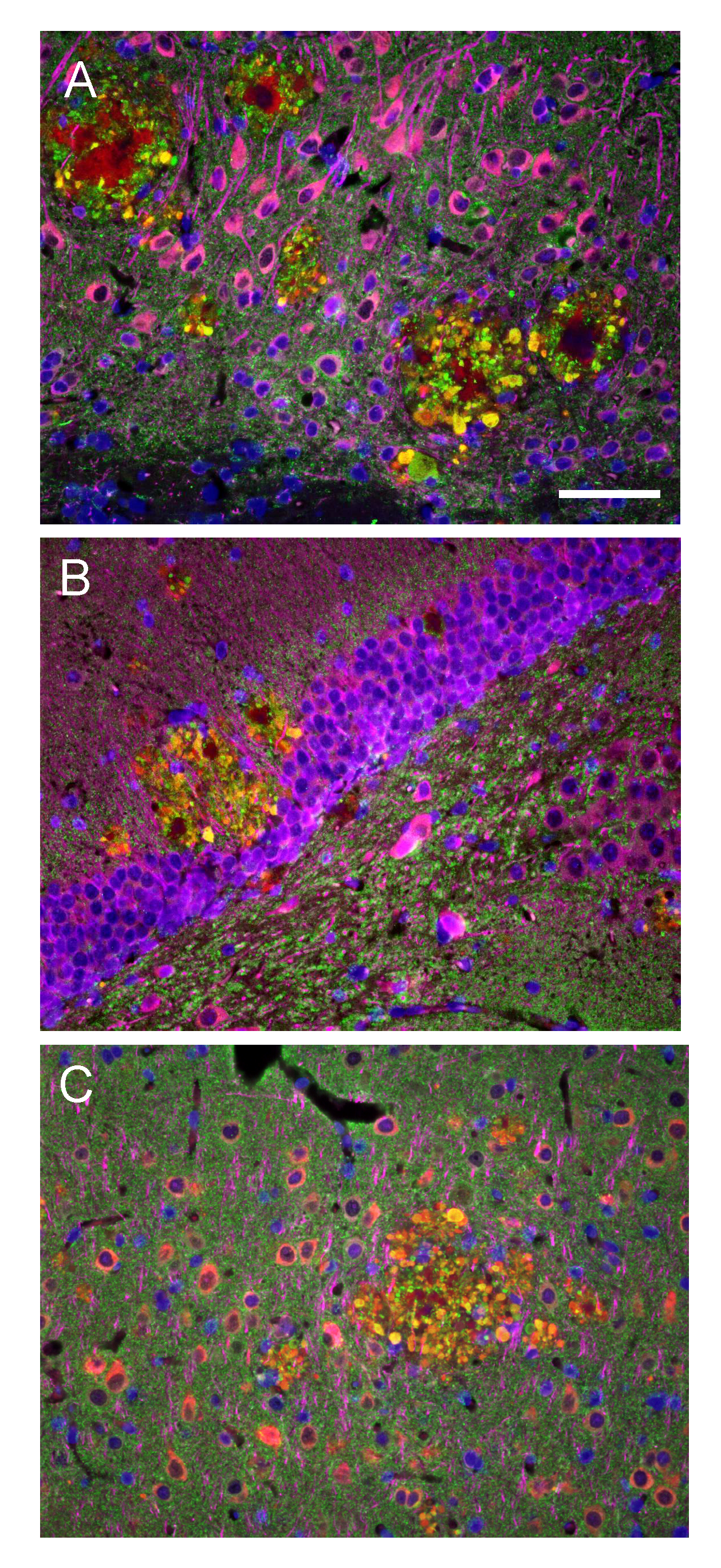

Supplement: Figure S5 — Triple-immunofluorescent detection of the pre-synaptic marker protein synaptophysin (green) accumulating in plaque-associated swollen dystrophic neurites of the plaque corona, apart from its physiological localization in the neuropil. Co-staining with the somato-dendritic marker MAP2 (magenta), with human APP (red) and nuclear staining with DAPI (blue). A, Subiculum of a 9 months-old hemizygous ARTE10 mouse. B, Dentate gyrus of a 7 months-old homozygous ARTE10 mouse. C, frontal cortex of an 8 months-old homozygous ARTE10 mouse. Scale bar for all three images, 50 µm. (10.00 MB TIF) [file pone.0007931.s006.tif]

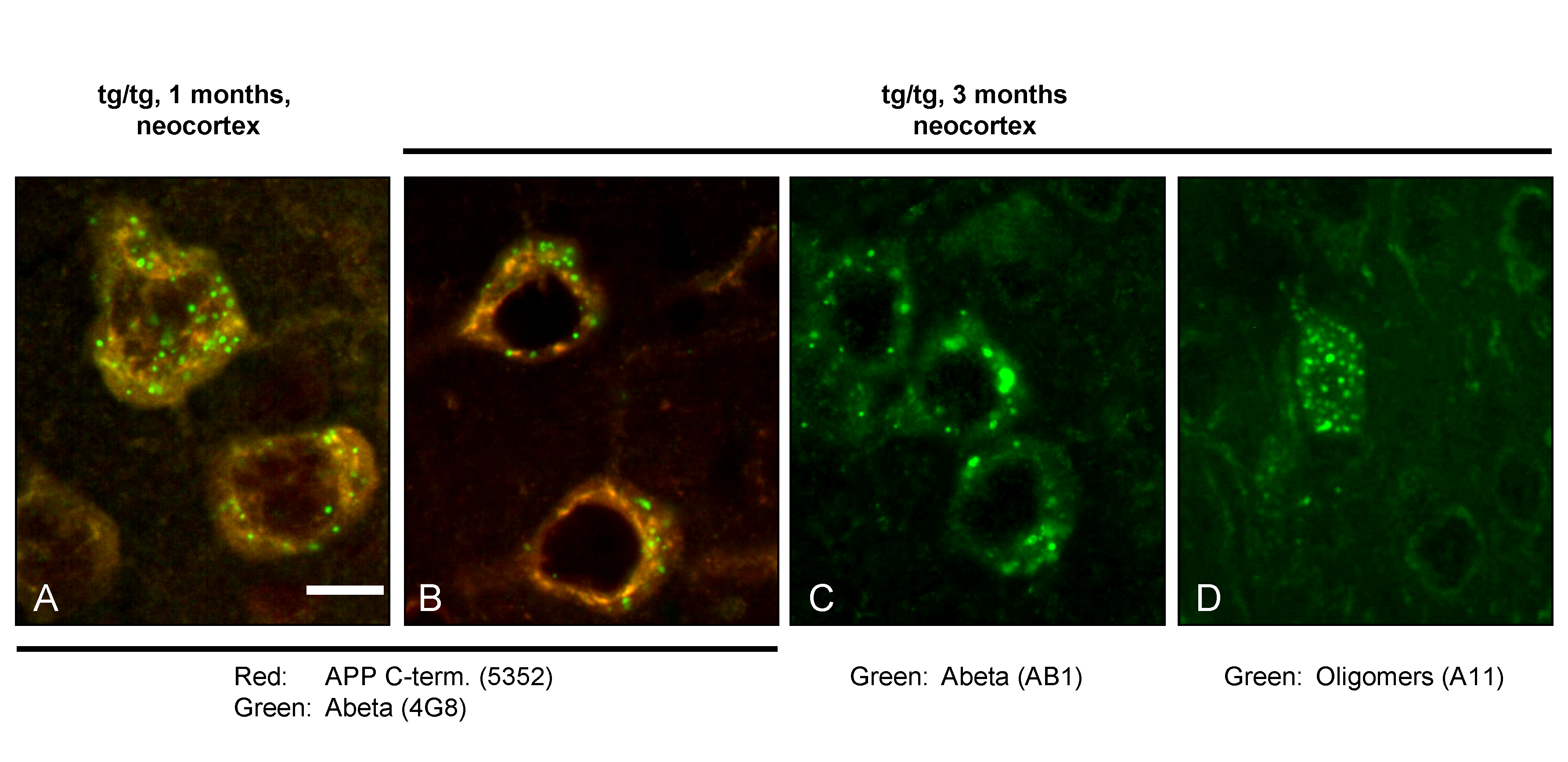

Supplement: Figure S6 — Immunofluorescent detection of intracellular Aβ (green) by 4G8 (A, B) or AB1 (C) in cortical neurons, shown here in two young ARTE10 animals (1 and 3 months). APP is co-stained in red in the left two images. The speckled intracytoplasmic Aβ immunostaining is paralleled by virtually the same pattern detected by the anti-oligomer conformation-specific antibody A11 (green, D) within the perikarya. This pattern closely resembles the intracellular Aβ immunoreactivity of affected neurons in human AD as well as in other murine AD models and suggests that intraneuronal Aβ is at least in part present in an oligomeric state early-on in ARTE10 mice. Scale bar in (A), 10 µm. (4.51 MB TIF) [file pone.0007931.s007.tif]

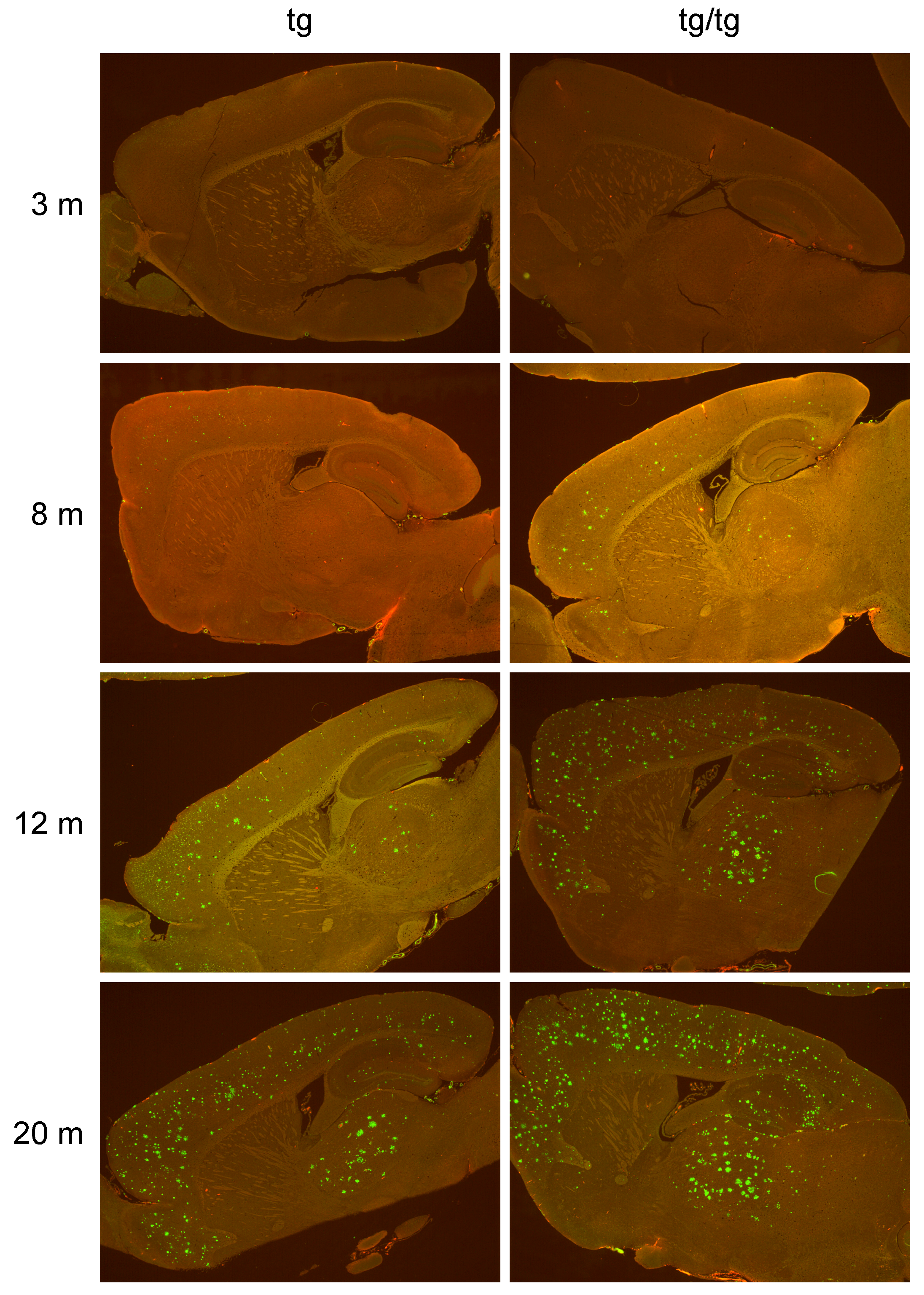

Supplement: Figure S7 — Thioflavin-S staining (green, FITC channel) for fluorescent detection of amyloid plaques in parasagittal brain sections of representative hemi- (tg) and homozygous (tg/tg) ARTE10 mice at different ages (3, 8, 12, and 20 months), illustrating the effects of age and gene dose on the plaque load. Tissue autofluorescence recorded in the red/Cy3 channel (in the absence of fluorophore) is utilized for the morphological visualization of gross anatomical structures. The sections are always oriented with the cortex at the top, the anterior part at the left, the posterior part at the right. Fluorescent micrographs were recorded separately for each channel by means of a Olympus BX51 microscope using the appropriate dichroic mirrors and filters, a 2x objective, a 0.5x TV adapter, and a ColorView-II CCD camera. The channels were merged using the Olympus analySIS “FIVE” imaging software. Parallel sections from the same animals as in Figure S8 are shown. (9.56 MB TIF) [file pone.0007931.s008.tif]

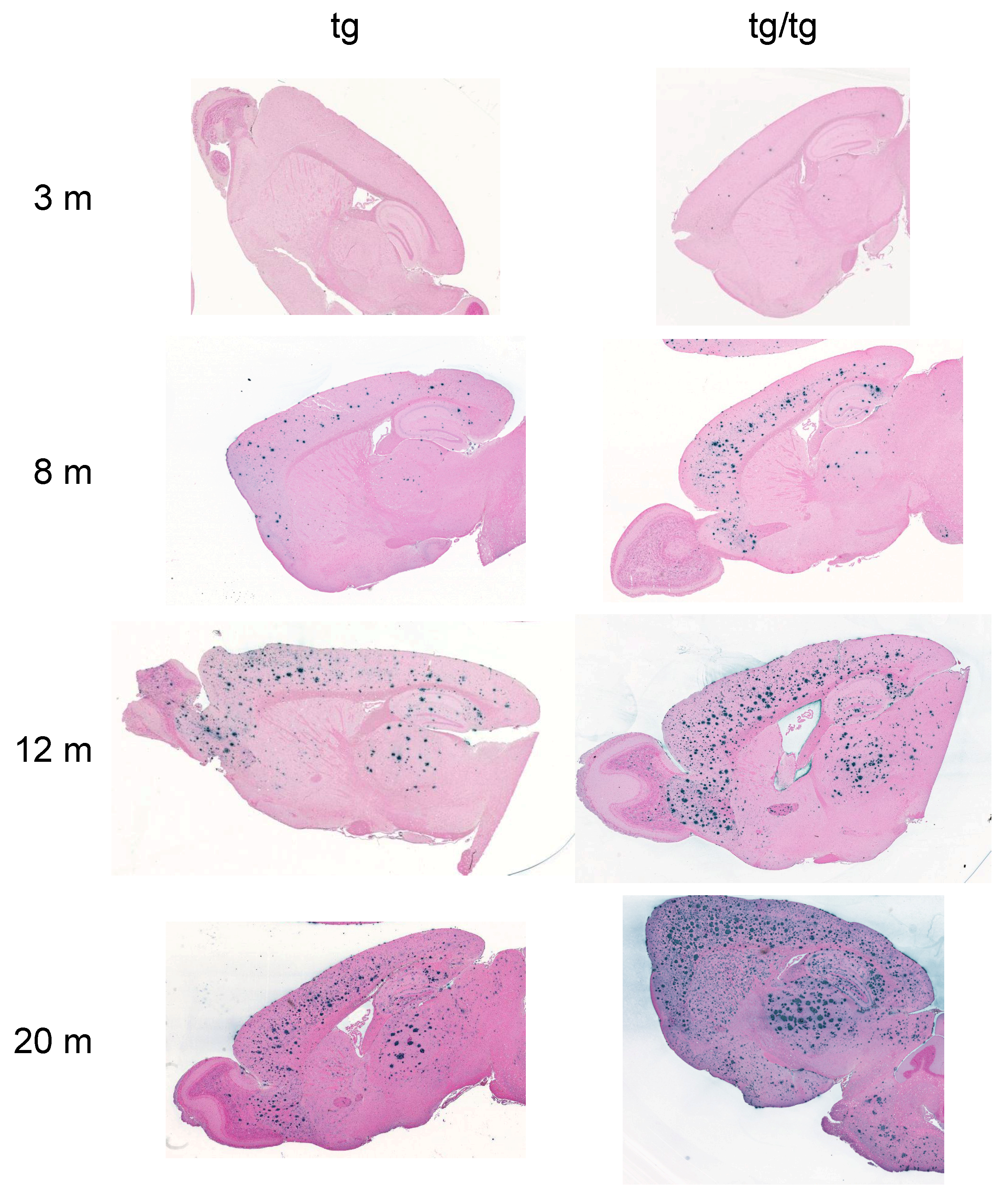

Supplement: Figure S8 — Immunohistochemical detection of Aβ (6E10, green) in parasagittal brain sections of representative hemi- (tg) and homozygous (tg/tg) ARTE10 mice at different ages (3, 8, 12, and 20 months), illustrating the effects of age and gene dose on the plaque load. (10.17 MB TIF) [file pone.0007931.s009.tif]

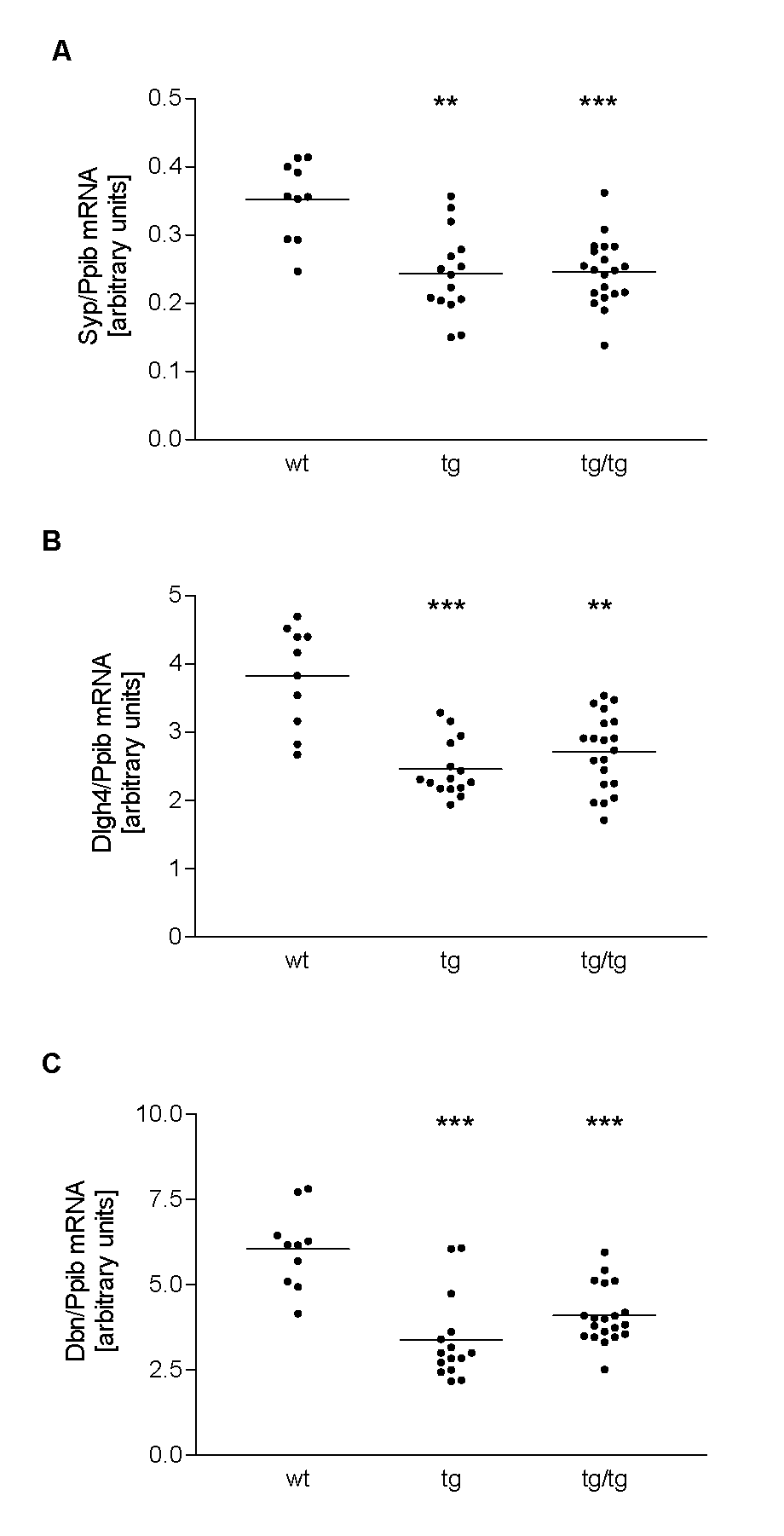

Supplement: Figure S9 — Statistical analysis of synaptic marker mRNA expression analysis. A, Synaptophysin (Syp), B, Disk large homolog 4 (Dlgh4), and C, Drebrin (Dbn1) mRNA expression in hemizygous and homozygous ART10 mice were analyzed in comparison to wild type animals. Material from comparable brain regions of mice ranging from 3 to 13 months of age was used for each of these studies. mRNA has been extracted from the brain material and expression of Synaptophysin, Disk large homolog 4, and Drebrin was measured applying real-time quantitative PCR. For normalization, each ratio of Synaptophysin, Disk large homolog 4, and Drebrin values with Cyclophilin B (Ppib) have been calculated. In comparison to wild type mice hemi- as well as homozygous mice revealed a significantly lower mRNA level of Synaptophysin (p = 0.0011; 0.0003), Disk large homolog 4 (p = 0.0003; 0.0011), and Drebrin (p = 0.0003; 0.0002), respectively; statistical analyses were done by Mann-Whitney U-test. (0.10 MB TIF) [file pone.0007931.s010.tif]
