# Supplementary material for: Early-Onset and Robust Amyloid Pathology in a New Homozygous Mouse Model of Alzheimer's Disease
Source: PLoS One. 2009 Nov 20;4(11):e7931. doi: 10.1371/journal.pone.0007931 (PMC2775952; doi:10.1371/journal.pone.0007931)
Supplement: Table S1 — Results of tests examining body weight, body temperature and motor-sensory abilities of hemizygous and homozygous transgenic mice in comparison to wild type littermates. Data are expressed as means +/− SEM; * p<0.05 versus wild type littermates (Wt); Tg hemizygous transgenic mice; Tg/tg homozygous transgenic mice. (0.01 MB PDF) [file pone.0007931.s011.pdf]

Age (month)		n	Body weight (g) females	Body weight (g) males	Body temperature (°C)	Grip strength (x 10 Nm)	Hot plate test (sec)	Rotarod females Trial 1 (sec)	Rotarod females Trial 2 (sec)	Rotarod females Trial 3 (sec)	Rotarod females Trial 4 (sec)	Rotarod males Trial 1 (sec)	Rotarod males Trial 2 (sec)	Rotarod males Trial 3 (sec)	Rotarod males Trial 4 (sec)
Longitudinal study															
4	Wt	15	20.8 ± 0.8	28.9 ± 0.9	38.0 ± 0.1	54 ± 2	34.9 ± 3.2	89 ± 17	115 ± 10	115 ± 17	129 ± 16	124 ± 20	101 ± 18	152 ± 22	182 ± 18
	Tg	16	21 ± 0.6	27.8 ± 0.8	37.8 ± 0.2	53 ± 2	31.6 ± 2.0	149 ± 20	138 ± 29	183 ± 35	205 ± 29	134 ± 20	108 ± 12	135 ± 23	140 ± 11
	Tg/tg	13	20.3 ± 0.5	26.4 ± 0.9	38.3 ± 0.1	53 ± 3	38.0 ± 2.9	126 ± 19	180 ± 26	177 ± 33	204 ± 19	125 ± 24	150 ± 36	151 ± 33	165 ± 48
8	Wt	15	23 ± 0.6	32.9 ± 1.4	38.2 ± 0.1	74 ± 4	27.8 ± 2.1	171 ± 24	158 ± 22	160 ± 21	169 ± 26	162 ± 19	166 ± 20	173 ± 19	186 ± 16
	Tg	16	23.3 ± 0.8	31.7 ± 0.9	37.8 ± 0.5	73 ± 3	31.6 ± 2.3	186 ± 22	226 ± 26	201 ± 30	203 ± 35	110 ± 16	156 ± 6	150 ± 20	170 ± 18
	Tg/tg	13	22.2 ± 0.6	31.1 ± 1.5	38.5 ± 0.1	71 ± 2	37.0 ± 2.8 *	188 ± 33	237 ± 21	251 ± 18	242 ± 21	164 ± 47	188 ± 43	223 ± 39	189 ± 49
12	Wt	15	23.5 ± 0.6	33.7 ± 1.6	37.4 ± 0.2	103 ± 5	30.2 ± 2.6	239 ± 32	203 ± 17	212 ± 30	197 ± 28	157 ± 15	199 ± 18	184 ± 15	181 ± 14
	Tg	16	25.1 ± 0.8	32.3 ± 0.9	37.7 ± 0.1	102 ± 4	34.9 ± 2.8	253 ± 21	237 ± 22	254 ± 17	238 ± 25	157 ± 20	128 ± 14	188 ± 17	176 ± 11
	Tg/tg	11	23.4 ± 0.7	30.2 ± 0.5	37.8 ± 0.2	100 ± 7	37.5 ± 5.2	256 ± 22	254 ± 23	257 ± 20	263 ± 18	195 ± 38	189 ± 29	182 ± 28	191 ± 14
Cross-sectional study															
8	Wt	14	26.3 ± 1.3	31.6 ± 1.2	37.9 ± 0.2	88 ± 6	29.3 ± 1.6	117 ± 17	162 ± 19	174 ± 14	181 ± 33	126 ± 21	133 ± 24	143 ± 19	173 ± 27
	Tg	15	24 ± 1.7	30.5 ± 0.6	37.7 ± 0.2	88 ± 5	34.2 ± 3.2	193 ± 12	187 ± 44	192 ± 37	225 ± 17	106 ± 8	106 ± 8	140 ± 17	135 ± 22
	Tg/tg	12	21.8 ± 1.2 *	31.7 ± 1.7	37.7 ± 0.2	92 ± 6	34.4 ± 1.2	157 ± 36	207 ± 37	153 ± 34	185 ± 41	100 ± 17	115 ± 14	111 ± 19	121 ± 13
12	Wt	14	24.4 ± 1.1	27 ± 2.4	37.4 ± 0.2	80 ± 4	35.9 ± 2.2	150 ± 31	156 ± 21	203 ± 26	174 ± 25	151 ± 30	110 ± 15	181 ± 29	207 ± 15
	Tg	14	24 ± 1.1	29.4 ± 1.6	37.8 ± 0.2	86 ± 5	40.6 ± 3.8	203 ± 29	185 ± 21	197 ± 25	215 ± 23	92 ± 15	107 ± 24	94 ± 10	143 ± 26
	Tg/tg	14	24.3 ± 0.4	33.8 ± 1.5	37.4 ± 0.2	87 ± 4	40.3 ± 3.6	157 ± 19	184 ± 26	196 ± 27	197 ± 22	89 ± 18	101 ± 12	102 ± 6	140 ± 20

* $p < 0.05$ versus wt
