# Supplementary material for: Early-Onset and Robust Amyloid Pathology in a New Homozygous Mouse Model of Alzheimer's Disease
Source: PLoS One. 2009 Nov 20;4(11):e7931. doi: 10.1371/journal.pone.0007931 (PMC2775952; doi:10.1371/journal.pone.0007931)
Supplement: Table S2 — Results of behavioural tests examining locomotor and exploratory activity, anxiety and spontaneous alternation of heterozygous and homozygous transgenic mice in comparison to wild type littermates. Data are expressed as means +/− SEM; * p<0.05 versus wild type littermates (Wt); # p<0.05 versus hemizygous littermates (Tg); Tg/tg homozygous transgenic mice. (0.01 MB PDF) [file pone.0007931.s012.pdf]

Age (month)		n	Open field: Locomotor activity (distance moved, m)	Open field: Exploratory activity (% time in explor. zone)	Zero maze: Entries in open sector (%)	Y maze: Arm entries (n)	Y maze: Spontaneous alternation (%)
Longitudinal study							
4	Wt	15	1.84 ± 0.16	47.7 ± 4.1	25.3 ± 6.1	31.1 ± 1.4	57.4 ± 2.1
	Tg	16	1.51 ± 0.14	40.4 ± 5.5	14.6 ± 4.0	24.4 ± 1.7 *	64.3 ± 2.4
	Tg/tg	13	1.66 ± 0.17	25.8 ± 4.1 *#	8.1 ± 2.3 *	22.8 ± 1.8 *	57.7 ± 3.1
8	Wt	15	1.77 ± 0.19	25.1 ± 4.4	6.1 ± 2.8	29.7 ± 2.4	60.3 ± 2.5
	Tg	16	1.51 ± 0.16	18.3 ± 3.4	5.6 ± 1.6	24.6 ± 1.5	61.0 ± 2.8
	Tg/tg	13	1.40 ± 0.16	18.0 ± 5.8	5.9 ± 1.7	20.3 ± 1.5 *	70.7 ± 4.2 *#
12	Wt	15	1.37 ± 0.15	14.7 ± 3.6	2.1 ± 0.8	22.5 ± 1.9	59.1 ± 2.8
	Tg	16	1.25 ± 0.12	11.6 ± 3.0	0.8 ± 0.5	22.1 ± 1.4	54.7 ± 3.2
	Tg/tg	11	1.32 ± 0.12	12.8 ± 2.9	0.9 ± 0.6	18.0 ± 1.2	55.9 ± 4.8
Cross-sectional study							
8	Wt	14	2.19 ± 0.19	38.3 ± 4.4	10.2 ± 2.6	26.2 ± 3.1	63.0 ± 2.5
	Tg	15	2.33 ± 0.13	39.7 ± 3.6	10.2 ± 1.9	18.5 ± 1.7 *	64.0 ± 1.9
	Tg/tg	12	1.75 ± 0.18 #	34.3 ± 5.1	9.0 ± 2.8	16.8 ± 2.0 *	65.0 ± 3.3
12	Wt	14	2.57 ± 0.30	45.0 ± 3.9	7.4 ± 3.4	29.6 ± 2.2	59.2 ± 2.3
	Tg	14	1.92 ± 0.14	53.5 ± 5.9	4.7 ± 1.9	19.9 ± 1.6 *	68.7 ± 3.1
	Tg/tg	14	2.05 ± 0.12	52.8 ± 5.1	15.3 ± 6.0	19.1 ± 1.5 *	68.1 ± 4.6

* $p<0.05$ versus wt
# $p<0.05$ versus tg
